# Synthesis of open-shell ladder π-systems by catalytic C–H annulation of diarylacetylenes†
†Electronic supplementary information (ESI) available: Syntheses, NMR, UV-vis-nearIR absorption, CV and crystallographic table. CCDC 1421865 (**1a**), 1421866 (**1b**). For ESI and crystallographic data in CIF or other electronic format see DOI: 10.1039/c5sc03391h


**DOI:** 10.1039/c5sc03391h

**Published:** 2015-10-22

**Authors:** Takehisa Maekawa, Hiroshi Ueno, Yasutomo Segawa, Michael M. Haley, Kenichiro Itami

**Affiliations:** a Graduate School of Science , Nagoya University , Chikusa , Nagoya 464-8602 , Japan . Email: ysegawa@nagoya-u.jp ; Email: itami@chem.nagoya-u.ac.jp; b JST , ERATO , Itami Molecular Nanocarbon Project , Chikusa , Nagoya 464-8602 , Japan; c Department of Chemistry & Biochemistry and Materials Science Institute , University of Oregon , Eugene , Oregon 97403-1253 , USA . Email: haley@uoregon.edu; d Institute of Transformative Bio-molecules (WPI-ITbM) , Nagoya University , Chikusa , Nagoya 464-8602 , Japan

## Abstract

A new open-shell ladder-shaped π-system has been synthesized. Pentaleno[1,2-*b*:4,5-*b*′]difluorene derivatives, 8 fused ring systems bearing 5- and 6-membered rings, were constructed from alkynylfluorenone through a reaction sequence including Pd-catalyzed C–H/C–H annulation. X-ray crystallography and ESR spectroscopy revealed the open-shell character of these ladder-shaped molecules, which derives from their extended π-electron conjugation. Absorption peaks in the near IR region as well as narrow redox potentials observed by cyclic voltammetry indicated small optical and fundamental energy gaps of these fused ring systems.

## Abstract 2

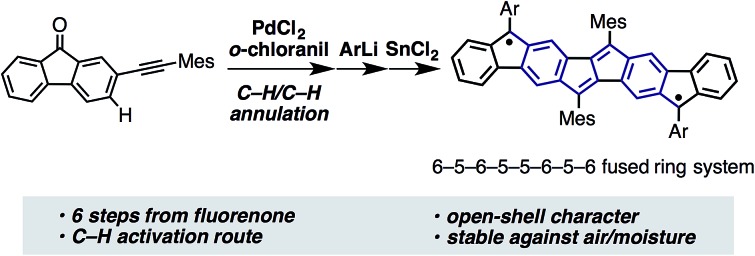
A new open-shell ladder-shaped π-system has been synthesized.

## Introduction

**Fig. 1:**
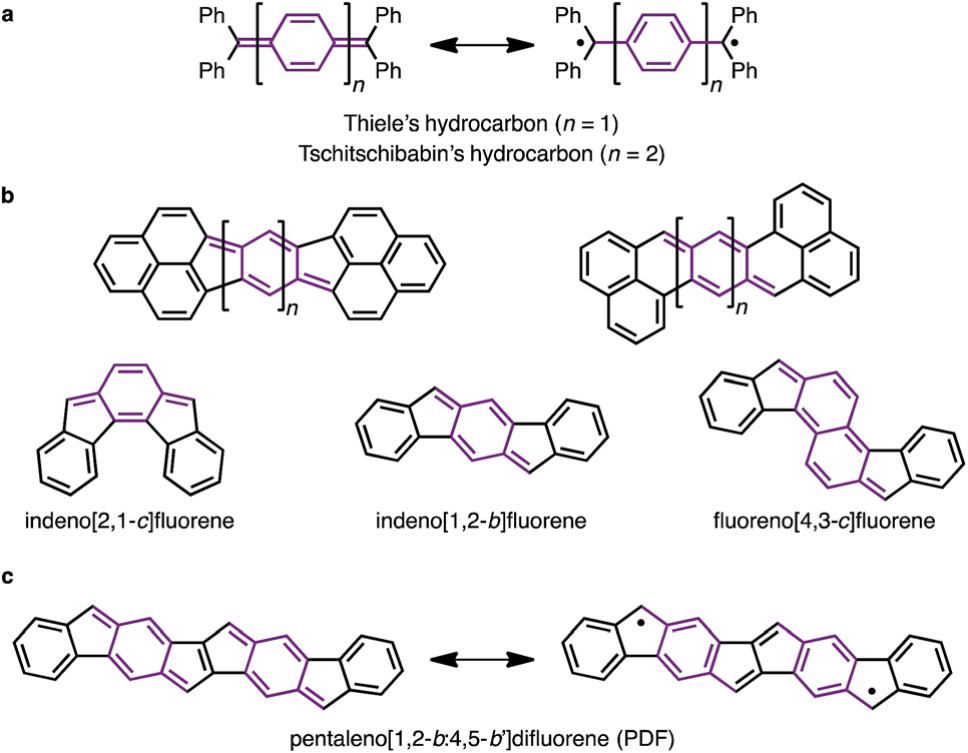
(a) Thiele's and Tschitschibabin's hydrocarbons. (b) Representative polycyclic conjugated hydrocarbons containing *p*-quinodimethane moieties. (c) PDF (this work). *p*-Quinodimethane moieties are highlighted in purple.

Since the discovery of Thiele's and Tschitschibabin's hydrocarbons (Fig. 1a),^1^ organic biradical compounds (open-shell π-systems) have attracted great interest from chemists, and more recently from materials scientists.^2^ Theoretical and experimental studies have revealed unique properties, such as a low energy gap, stimuli-responsive spin structure, and singlet fission.^2^–^4^ Despite their practical utility, however, high chemical reactivity and short lifetimes are common concerns in radical chemistry and in device applications. Among the various types of fused ring systems, linearly extended polycyclic structures bearing *p*-quinodimethane moieties (Fig. 1b) are found in either open-shell (phenalenyls) or closed-shell (indenyl) carbon-based π-systems depending upon the nature of the ring fusion.^3^,^4^ Although significant progress, such as C–H activation reactions,^5^ has been made toward the synthesis of novel polycyclic structures, the construction of extended carbon-based π-systems still represents a challenge.5*b* To push the chemistry of open-shell conjugated hydrocarbons forward, more powerful synthetic methods and a novel structural design are needed. Herein, we report the facile synthesis of a novel carbon-based π-system having 8 fused rings, pentaleno[1,2-*b*:4,5-*b*′]difluorene (PDF), which has possible resonance structures of closed shell and diradical as shown in Fig. 1c. Aryl-substituted PDFs were synthesized from simple alkynylfluorenone by applying our recently reported C–H activation reaction.^6^ The NMR, ESR, absorption, and CV measurements of PDF revealed their open-shell character with narrow HOMO–LUMO energy gaps.

## Results and discussion

### DFT study of PDF

**Fig. 2:**
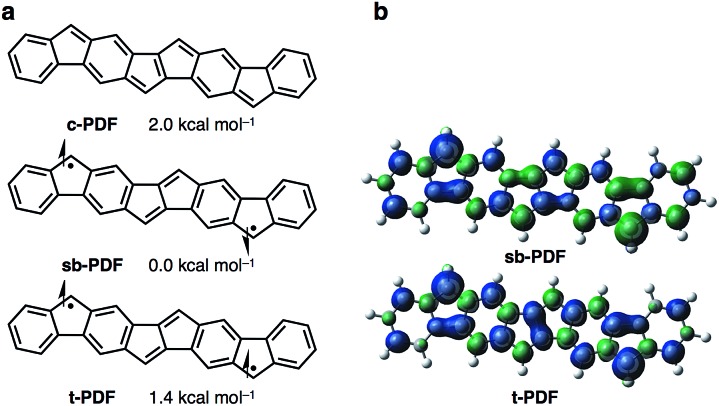
(a) Electronic states of PDF with energy values relative to the most stable states. (b) Spin density distributions. Blue and green surfaces represent *α* and *β* spin density, respectively. Isovalue is 0.003. Calculated at the (U)B3LYP/6-311+G(d,p) level.

We began by performing a density functional theory (DFT) study on the electronic states of neutral PDF in order to estimate the physical properties of the target compounds. The structures of unsubstituted PDF was optimized at the (U)B3LYP/6-311+G(d,p) level. Fig. 2a shows the possible electronic states of PDF. Three electronic states, closed-shell (**c-PDF**), open-shell singlet biradical (**sb-PDF**), and triplet (**t-PDF**), were found. The relative energy values of the three electronic states indicated **sb-PDF** as the most favorable at the ground state, whereas **c-PDF** and **t-PDF** are slightly higher in energy (2.0 and 1.4 kcal mol^–1^, respectively).^6^ The biradical character (*y*) of **sb-PDF** estimated from the natural orbital occupancy number (NOON)^7^ of the lowest unoccupied natural orbital (LUNO) by B3LYP/6-311+G(d,p) method is 55%. In the open-shell structures, **sb-PDF** and **t-PDF**, spin densities are delocalized throughout the molecules and the highest densities were found at the C9-position of the fluorene moieties, as illustrated in Fig. 2b. Based on these theoretical studies, PDF is expected to exhibit open-shell character, and PDF may show singlet–triplet transition.

### Synthesis of Ar_4_PDF (**1a**, **b**)

**Fig. 3:**
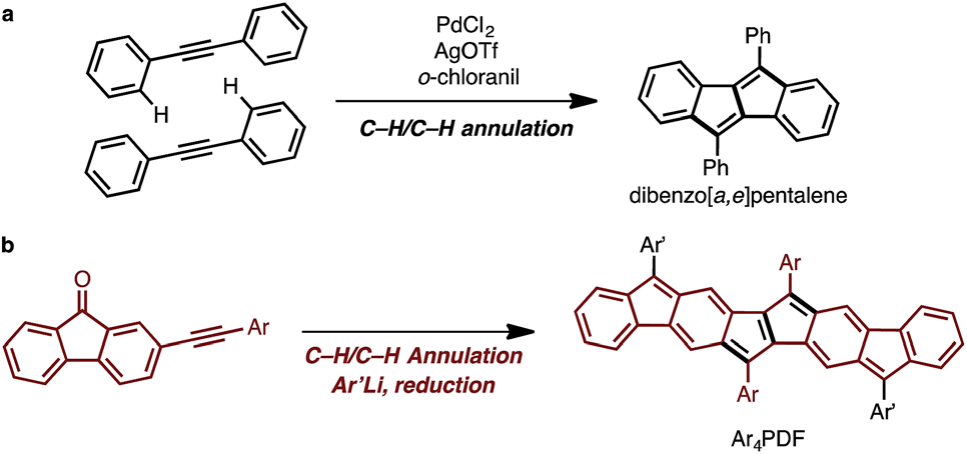
(a) Pd-catalyzed oxidative C–H/C–H annulation for the synthesis of dibenzo[*a*,*e*]pentalene. (b) Strategy for the synthesis of Ar_4_PDF.

The synthetic strategy for Ar_4_PDF is shown in Fig. 3. We have previously developed a Pd-catalyzed oxidative C–H/C–H annulation of diarylacetylenes^8^ to form dibenzo[*a*,*e*]pentalene frameworks in the presence of PdCl_2_/AgOTf/*o*-chloranil^9^ (Fig. 3a). We thus envisaged that elongated ladder-shaped frameworks could be easily constructed from alkynylfluorenones using the C–H/C–H annulation reaction (Fig. 3b). The thus-formed pentalene-fused fluorenones could then be converted into Ar_4_PDF by sequential nucleophilic addition of an aryllithium and reductive dehydroxylation, a commonly used method for the synthesis of quinoidal hydrocarbons.^4^

**Scheme 1:**
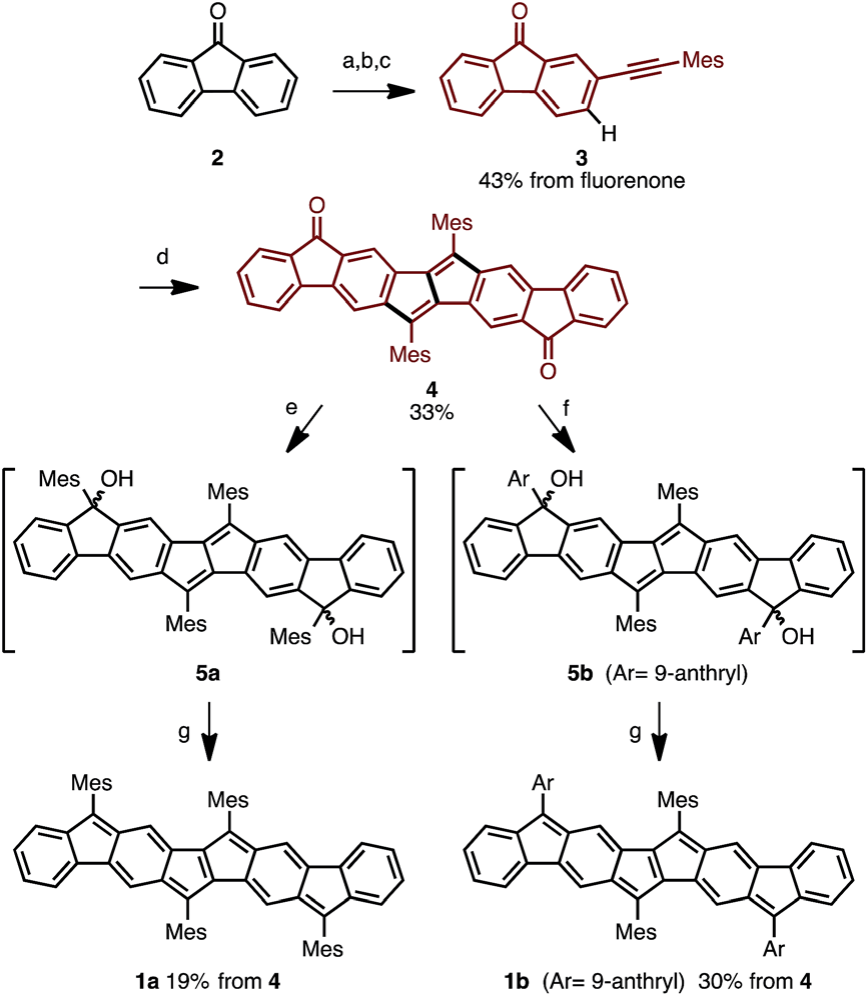
Synthesis of ladder-shaped open-shell molecules **1a**, **b**. Reaction conditions: (a) Br_2_, H_2_O, 80 °C, 4 h; (b) Me_3_SiCCH, PdCl_2_(PPh_3_)_2_, CuI, THF, NEt_3_, reflux, 9 h; (c) MesI, PdCl_2_(PPh_3_)_2_, CuI, H_2_O, DBU, benzene, 80 °C, 6 h; (d) PdCl_2_, *o*-chloranil, AgOTf, DMAc, 80 °C, 18 h; (e) MesLi, THF, –78 °C, 1.5 h; (f) 9-anthrylLi, THF, –78 °C, 1.5 h; (g) SnCl_2_, toluene, r.t., 4 h. Mes = 2,4,6-trimethylphenyl, DMAc = *N*,*N*-dimethylacetamide.

Following this strategy, Ar_4_PDF (**1a** and **1b**) were prepared in six steps from fluorenone (**2**), as shown in Scheme 1. To begin with, 2-(mesitylethynyl)fluorenone (**3**), which can be obtained by mono-bromination of fluorenone^10^ and subsequent Sonogashira cross-coupling,^11^ was subjected to Pd-catalyzed C–H/C–H annulation. The mesityl (2,4,6-trimethylphenyl) group was chosen for two reasons: (i) an aryl group with no *ortho*-C–H bond is required for the selective cleavage of fluorenone C–H bond; and (ii) the bulky mesityl group can stabilize the reactive pentalene moiety. Gratifyingly, the C–H cleavage proceeded selectively at the C3-position of **3** to afford dione **4** in 33% yield as a brown solid. Treatment of **4** with mesityllithium or 9-anthryllithium successfully gave diols **5a** and **5b**, respectively, as a mixture of diastereomers. After removal of unreacted starting materials, the diols **5** were mixed with excess SnCl_2_ at room temperature to produce Ar_4_PDFs **1a** and **1b** bearing the [6-5-6-5-5-6-5-6] fused ring systems. The products were obtained as deep green solids regardless of the aryllithium reagent used. It should be noted that **1a** showed high durability against air exposure for several months in solid state, whereas **1b** decomposed slowly under air. The quinoidal molecules **1a** and **1b** could even be isolated by silica-gel column chromatography in air. The *m*/*z* values and isotope patterns in the high-resolution mass spectra of these products were fully consistent with the calculated values.

### X-ray structures of **1a** and **1b**

**Fig. 4:**
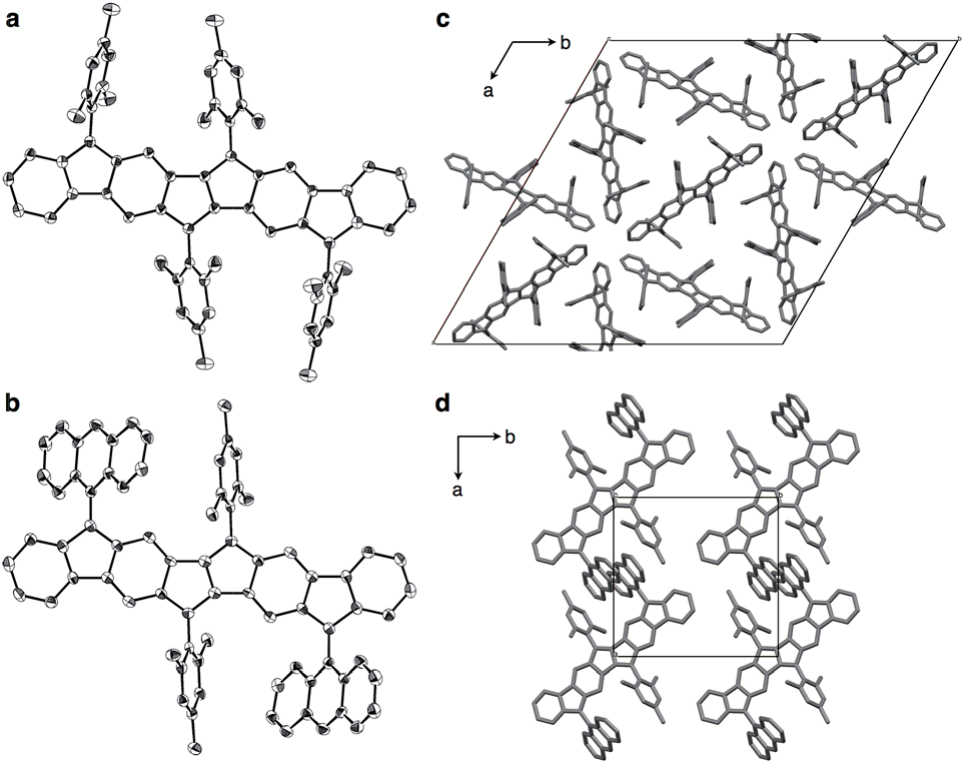
ORTEP drawings of **1a** (a) and **1b** (b) with 50% probability (all hydrogen atoms and solvent molecules are omitted for clarity; half of the entire structure constitutes an asymmetric unit), and packing structure of **1a** (c) and **1b** (d) along *c* axis.

Compounds **1a** and **1b** could be crystallized from CS_2_/Et_2_O and their exact molecular structures were unambiguously confirmed by X-ray diffraction (Fig. 4). Dihedral angles between PDF planes and the outer aryl groups on the fluorene moieties were 74.8° (**1a**) and 57.3° (**1b**), respectively, reflecting the bulkiness of the aryl groups. In the packing structures of **1a** and **1b**, no intermolecular π–π stacking of the PDF core was found. The 9-anthryl moieties of **1b** were stacked intermolecularly as shown in Fig. 4d.

Table 1 summarizes the bond lengths of **1a** and **1b**, and unsubstituted PDFs optimized at the B3LYP/6-311+G(d,p) level of theory. Despite different aryl groups (mesityl or 9-anthryl) being attached to the fluorene moieties, no significant difference was found in the bond lengths and angles of the PDF moiety in **1a** and **1b**, besides a small difference in bond i. Bonds i–v are double bonds in the closed-shell structure (**c-PDF**), whereas, in the open-shell structures (**sb-PDF** and **t-PDF**), three bonds (i, iv, and v) are single bonds and two bonds (ii and iii) are conjugated bonds (bond order ∼ 1.5). The small difference in the bond lengths between **1a** and **1b** indicated that the effect of the packing modes were negligible in this case. Clearly, the five bonds of **sb-PDF** and **t-PDF** are longer than those of **c-PDF**, especially i, iv, and v. In all bonds, little differences are observed between **sb-PDF** and **t-PDF**. The bond lengths of **1a** and **1b** observed by X-ray crystallography at 103 K were between those of open-shell (**sb-PDF** and **t-PDF**) and closed-shell (**c-PDF**), and rather close to the open-shell structures (see also Table S2 in ESI† for detail). We concluded that the PDF core structure of **1a** and **1b** is a hybrid of the closed shell and open shell forms in the solid state.

**Table 1:** Selected bond lengths of PDFs **1a**, **b**, and calculated analogues (Å)

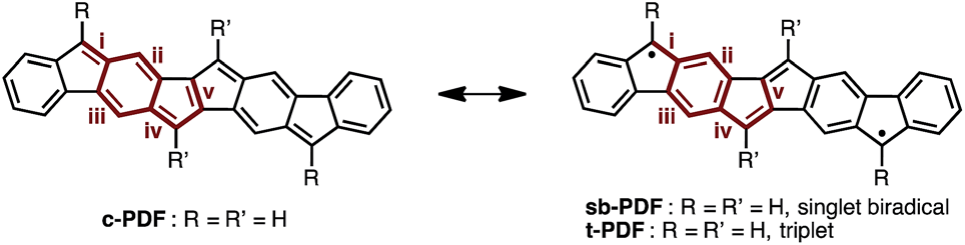					
	**1a**	**1b**	**c-PDF** ^*a*^	**sb-PDF** ^*a*^	**t-PDF** ^*a*^
i	1.401(3)	1.416(6)	1.388	1.410	1.410
ii	1.363(3)	1.364(6)	1.370	1.378	1.381
iii	1.372(3)	1.374(7)	1.374	1.388	1.390
iv	1.424(3)	1.420(6)	1.407	1.445	1.457
v	1.407(5)	1.415(9)	1.417	1.453	1.476

^*a*^Optimized at the (U)B3LYP/6-311+G(d,p) level.

### NMR, ESR, and SQUID measurement of **1a**

**Fig. 5:**
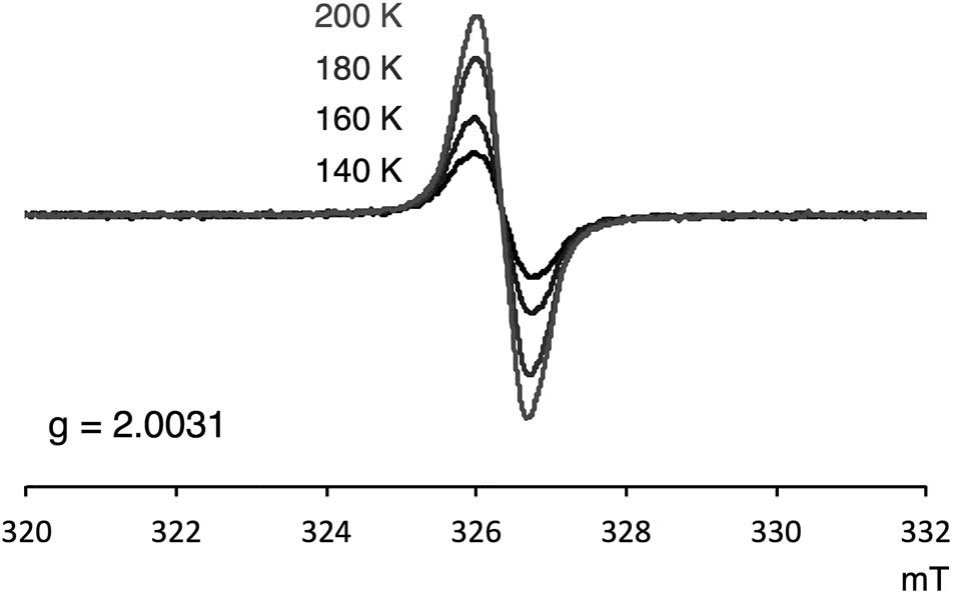
VT ESR spectra of **1a** in solid state.

Several experimental measurements of **1a** and **1b** strongly implicated their open-shell character. For example, broad signals in the ^1^H NMR spectra were observed, which clearly suggests the paramagnetic character of these compounds. Only three singlets, which can be assigned to the mesityl group,^12^ were observed in the ^1^H NMR spectra of both **1a** and **1b**. Even at –90 °C, the ^1^H NMR signals of **1a** were still broad (slightly sharper) thus indicating appreciable triplet character (see Fig. S4 in ESI†). ESR was also measured to confirm the open-shell properties of PDF. As shown in Fig. 5, the powder solid of **1a** exhibited ESR signals with no obvious spin–spin coupling probably due to the long spin–spin distances and no intermolecular stacking. The *g* values of **1a** was 2.0031, which are in the range of typical carboradical species.^2^ The singlet ground state of **1a** was indicated by VT ESR and preliminary SQUID measurements. The ESR signal of **1a** decreased as temperature decreased (Fig. 5), and the temperature dependence of magnetization obtained from SQUID measurement also supported singlet–triplet transition of **1a**. From the result of SQUID measurement, singlet–triplet energy gap (Δ*E*_S–T_) could be estimated to 3.4 kcal mol^–1^ (see ESI† for detail).

### UV-vis-near-IR absorption spectra and CV measurement

**Fig. 6:**
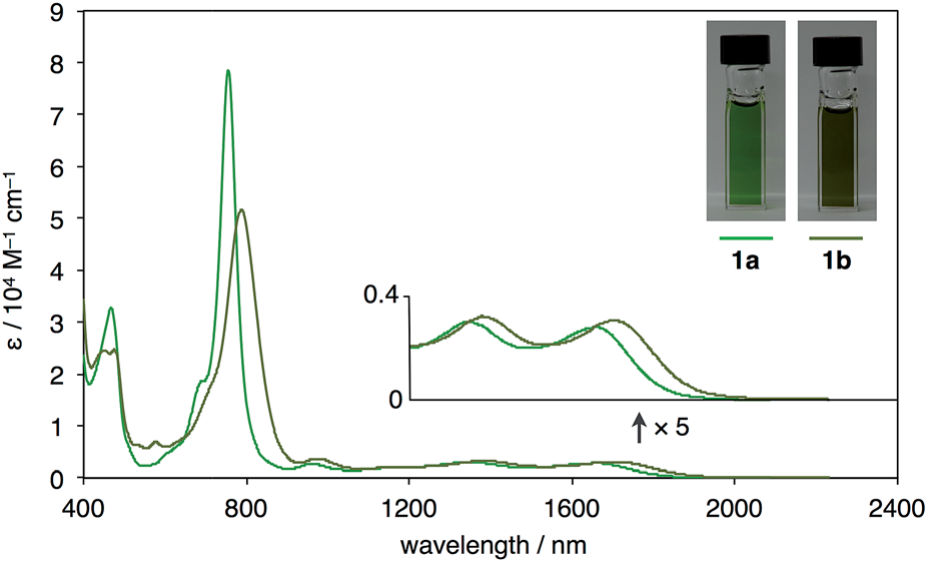
UV-vis-NIR absorption spectra and photographs of CS_2_ solution (*ca.* 1 × 10^–4^ M) of **1a** and **1b**.

**Fig. 7:**
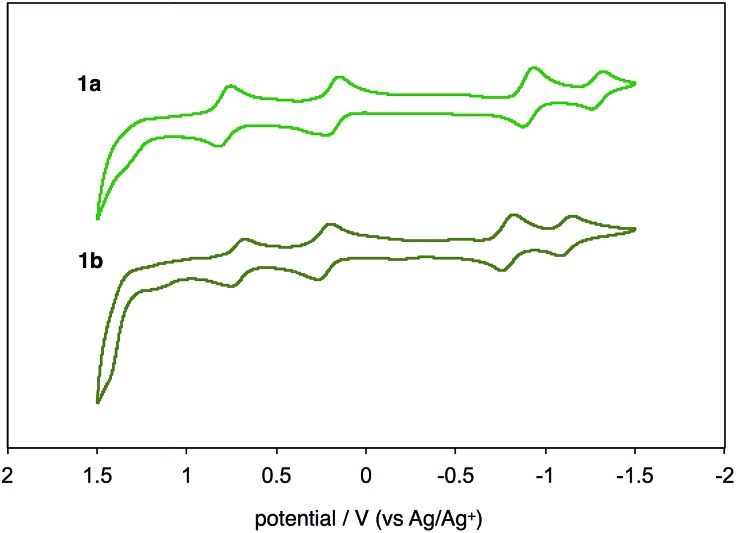
Cyclic voltammograms of **1a** and **1b** in CH_2_Cl_2_ with *n*-Bu_4_N^+^(CF_3_SO_2_)_2_N^–^ as supporting electrolyte, and scan rate of 0.1 V s^–1^.

UV-vis-near-IR absorption spectra as well as cyclic voltammogram (CV) proved small energy level gaps derived from open-shell states. Compounds **1a** and **1b** exhibit their highest absorption peaks centered at 756 and 786 nm, respectively, along with three bands in the near-IR region (Fig. 6). The lowest energy absorption bands at 1652 nm (**1a**) and 1702 nm (**1b**) can be seen. Cyclic voltammetry of **1a** and **1b** were also measured (Fig. 7). Two oxidation steps and two reduction steps were clearly observed in the CV chart of **1a** and **1b**. From CV results, fundamental energy gaps can be roughly estimated to 0.75 eV (**1a**) and 0.73 eV (**1b**), which are very close to the values obtained from the near-IR absorption peaks.

## Conclusions

We have achieved the rapid synthesis of linearly elongated, hexagon/pentagon-fused π-systems (pentaleno[1,2-*b*:4,5-*b*′]difluorene derivatives) by Pd-catalyzed C–H/C–H annulation. The synthesized molecules exhibited open-shell properties with small energy gap, which were confirmed by ESR spectra, NIR absorption, and CV. Although there is still room for improvement in the efficiency of key reactions, the successful rapid access to previously untapped molecular structures speaks well for the significant opportunities of C–H activation reactions in the chemistry of organic radical materials. Theoretical studies as well as further experimental investigations of the obtained open-shell π-systems are ongoing in our laboratory.

## Supplementary Material

Supplementary information

Crystal structure data

## References

[cit1] Thiele J., Balhorn H. (1904). Chem. Ber..

[cit2] Sun Z., Wu J. (2012). J. Mater. Chem..

[cit3] Ohashi K., Kubo T., Masui K., Yamamoto K., Nakatsuji K., Takui T., Kai Y., Murata I. (1998). J. Am. Chem. Soc..

[cit4] Chase D. T., Fix A. G., Rose B. D., Weber C. D., Nobusue S., Stockwell C. E., Zakharov L. N., Lonergan M. C., Haley M. M. (2011). Angew. Chem., Int. Ed..

[cit5] Wencel-Delord J., Glorius F. (2013). Nat. Chem..

[cit6] We also calculated by (U)CAM-B3LYP/6-311+G(d,p) and (U)M06-2X/6-311+G(d,p). See ESI for detail

[cit7] Döhnert D., Koutecký J. (1980). J. Am. Chem. Soc..

[cit8] Maekawa T., Segawa Y., Itami K. (2013). Chem. Sci..

[cit9] Mochida K., Kawasumi K., Segawa Y., Itami K. (2011). J. Am. Chem. Soc..

[cit10] Zhang X., Han J., Li P.-F., Ji X., Zhang Z. (2009). Synth. Commun..

[cit11] Sonogashira K., Tohda Y., Hagihara N. (1975). Tetrahedron Lett..

[cit12] Peak assignment was supported by calculation of the ^1^H NMR chemical shift of **1a** by using GIAO B3LYP/6-311+G(2d,p)//B3LYP/6-31G(d) level of theory. See ESI for detail

