# Supplementary material for: Synthesis of open-shell ladder π-systems by catalytic C–H annulation of diarylacetylenes
Source: Chem Sci. 2015 Oct 22;7(1):650–4. doi: 10.1039/c5sc03391h (PMC5953004; doi:10.1039/c5sc03391h)

Electronic Supplementary Material (ESI) for Chemical Science.
This journal is © The Royal Society of Chemistry 2015

Supporting Information

Synthesis of Open-Shell Ladder π-Systems by Catalytic C–H Annulation of Diarylacetylenes

Takehisa Maekawa,[1] Hiroshi Ueno,[1,2] Yasutomo Segawa,[1,2,*]
Michael M. Haley,[3,*] and Kenichiro Itami[1,2,4,*]

[1]*Graduate School of Science, Nagoya University, Chikusa, Nagoya 464-8602, Japan.*
[2]*JST, ERATO, Itami Molecular Nanocarbon Project, Chikusa, Nagoya 464-8602, Japan.*
[3]*Department of Chemistry and Materials Science Institute, University of Oregon, Eugene, Oregon 97403-1253, United States.*
[4]*Institute of Transformative Bio-molecules (WPI-ITbM), Nagoya University, Chikusa, Nagoya 464-8602, Japan.*

E-mail: ysegawa@nagoya-u.jp (YS), haley@uoregon.edu (MMH), itami@chem.nagoya-u.ac.jp (KI)

Table of Contents

1. Experimental section S1–S7
2. X-ray crystallography S8–S10
3. VT NMR spectroscopy S11
4. UV–vis–NIR absorption spectroscopy S12
5. Cyclic voltammetry S13
6. SQUID measurement S14
7. Computational study S15–S16
8. ^{1}H and ^{13}C NMR spectra of new compounds S17–S24

1. Experimental section

General

Unless otherwise noted, all reagents including anhydrous solvents were obtained from commercial suppliers and used without further purification. 2-bromofluorenone (**8**) was prepared according to literature procedure.[S1] AgOTf was stored in a glovebox filled by argon prior to use. *o*-Chloranil was recrystallized from benzene prior to use. Column chromatography was performed with silica-gel 60 (230–400 mesh). Synthetic manipulations that required an inert atmosphere (where noted) were carried out under nitrogen using standard Schlenk techniques or in an inert-atmosphere glove box. Analytical thin-layer chromatography (TLC) was performed using E. Merck silica-gel 60 F_{254} precoated plates (0.25 mm). The developed chromatogram was analyzed by UV lamp (254 nm and 365 nm), ethanolic phosphomolybdic acid. High-resolution mass spectra (HRMS) were obtained from a JMS-T100TD instrument (DART), Thermo Fisher Scientific Exactive (APCI). Nuclear magnetic resonance (NMR) spectra were recorded on a JEOL ECA-600 (^{1}H NMR 600 MHz, ^{13}C NMR 150 MHz) spectrometers or a JEOL ECA-400 (^{1}H NMR 400 MHz, ^{13}C NMR 100 MHz). Chemical shifts for ^{1}H NMR are expressed in parts per million (ppm) relative to solvent signal ^{1}H ($CHCl_3$: 7.26 ppm; $CHDCl_2$: 5.30 ppm) and ^{13}C ($CDCl_3$: 77.0 ppm; CD_2Cl_2: 53.8 ppm). Data are reported as follows: chemical shift, multiplicity (s = singlet, d = doublet, dd = doublet of doublets, t = triplet, td = triplet of doublet, m = multiplet, br = broadening signal).

S1) X. Zhang, J. Han, P.-F. Li, X. Ji and Z. Zhang, *Synth. Commun.* **2009**, 3804.

Synthesis of 9

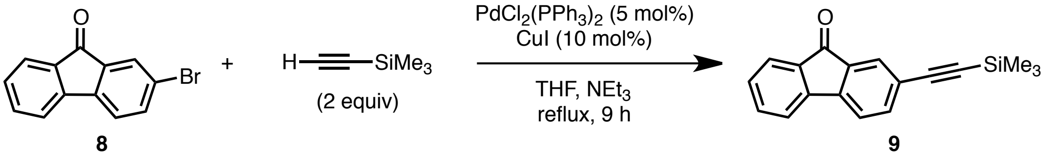

To a heat-dried 100-mL two necked round bottom flask were added a magnetic stirring bar, **8** (2.76 g, 10.0 mmol), $PdCl_2(PPh_3)_2$ (361 mg, 500 μmol) and CuI (190 mg, 1.00 mmol). Contents were evacuated, then back-filled with nitrogen gas three times. Then, dry THF (20 mL), ethynyltrimethylsilane (2.9 mL, 20 mmol) and NEt_3 (20 mL) were added. The resulting mixture was stirred at 80 °C for 9 hours. After cooling to room temperature, saturated NH_4Cl aqueous solution was added. The organic layer extracted with CH_2Cl_2, washed with brine, dried over Na_2SO_4 and the solvents were evaporated under reduced pressure to afford the crude reaction mixture. The crude reaction mixture was purified by silica-gel column chromatography (hexane/CH_2Cl_2 = 2:1) to afford product **9** as a yellow solid (3.20 g, 85%).

^{1}H NMR (600 MHz, $CDCl_3$) δ 0.26 (s, 9H), 7.31 (td, *J* = 7.2, 1.4 Hz, 1H), 7.47 (d, *J* = 7.9 Hz, 1H), 7.49–7.53 (m, 2H), 7.58 (dd, *J* = 7.9, 1.4 Hz, 1H), 7.67 (d, *J* = 7.3 Hz, 1H), 7.74 (s, 1H); ^{13}C NMR (150 MHz, $CDCl_3$) δ –0.1, 96.3, 104.0, 120.1, 120.6, 124.1, 124.5, 127.7, 129.4, 134.1, 134.4, 134.9, 138.0, 143.91, 143.93, 193.0; HRMS (DART) *m*/*z* calcd for $C_{18}H_{16}OSi$ $[M+H]^+$: 277.10487, found: 277.10472.

Synthesis of 3

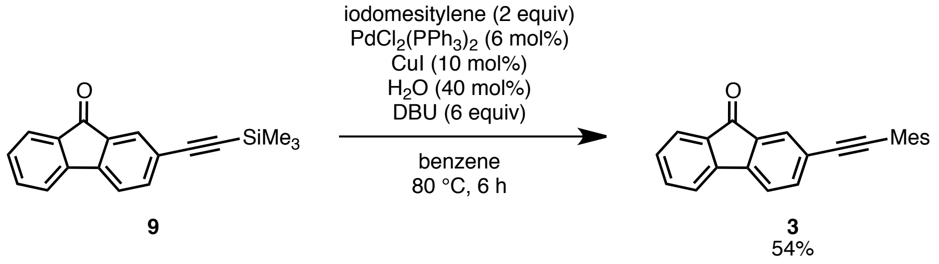

To a heat-dried 300-mL two necked round bottom flask were added a magnetic stirring bar, **9** (3.50 g, 12.0 mmol), iodomesitylene (6.40 g, 25.0 mmol), $PdCl_2(PPh_3)_2$ (361 mg, 800 μmol) and CuI (250 mg, 1.30 mmol). Contents were evacuated, then back-filled with nitrogen gas three times, then dry benzene (65 mL), H_2O (90 μL, 5.0 mmol) and 1,8-diazabicyclo[5.4.0]undec-7-ene (12 mL) were added. The resulting mixture was stirred at 80 °C for 6 hours. After cooling to room temperature, saturated NH_4Cl aqueous solution was added. The organic layer extracted with CH_2Cl_2, washed with brine, dried over Na_2SO_4 and the solvents were evaporated under reduced pressure to afford the crude reaction mixture. The crude reaction mixture was purified by silica-gel column chromatography (hexane/CH_2Cl_2 = 3:1) to afford product **3** as a yellow solid (2.20 g, 54%).

^{1}H NMR (600 MHz, $CDCl_3$) δ 2.30 (s, 3H), 2.48 (s, 6H), 6.91 (s, 2H), 7.32 (td, J = 7.3, 1.3 Hz, 1H), 7.508 (d, J = 7.6 Hz, 1H), 7.512 (td, J = 7.3, 1.3 Hz, 1H), 7.54 (d, J = 7.2 Hz, 1H), 7.63 (dd, J = 7.6, 1.4 Hz, 1H), 7.68 (d, J = 7.2 Hz, 1H), 7.80 (d, J = 1.0 Hz, 1H); ^{13}C NMR (150 MHz, $CDCl_3$) δ 21.0, 21.4, 89.2, 96.3, 119.5, 120.3, 120.5, 124.5, 124.9, 127.0, 127.7, 129.3, 134.2, 134.3, 134.9, 137.2, 138.2, 140.3, 143.3, 144.1, 193.3; HRMS (DART) m/z calcd for $C_{24}H_{18}O$ $[M+H]^+$: 323.14359, found: 323.14340.

Synthesis of 4

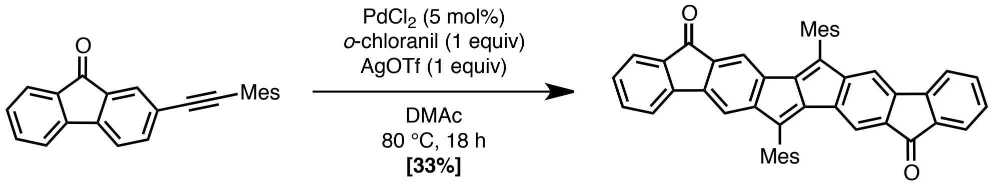

To a heat-dried screw-capped test tube were added a magnetic stirring bar, **3** (756 mg, 2.34 mmol), $PdCl_2$ (21.0 mg, 117 μmol) and *o*-chloranil (575 mg, 2.34 mmol). The screw-capped test tube was introduced inside an argon atmosphere glovebox. To the tube was added AgOTf (601 mg, 2.34 μmol). The screw-capped tube was taken out of the glovebox, and to it was added anhydrous DMAc (12 mL). The test tube was sealed and the resulting mixture was stirred at 80 °C for 18 hours. After cooling to room temperature, contents were diluted with $CHCl_3$. The organic layer was passed on a short silica plug, eluted with $CHCl_3$. The solvents were evaporated under reduced pressure to afford the crude reaction mixture. The crude reaction mixture was extracted by $CHCl_3$ twice to remove residual DMAc and purified by silica-gel column chromatography (hexane/CH_2Cl_2 = 1:1). The obtained product was carefully washed and filtered by MeOH for further purification and the purified product was afforded as a brown solid (250 mg, 33%).

^{1}H NMR (600 MHz, $CDCl_3$) δ 2.30 (s, 12H), 2.43 (s, 6H), 6.70 (s, 2H), 6.86 (s, 2H), 7.06 (s, 4H), 7.23 (t, *J* = 7.2 Hz, 2H), 7.31 (d, *J* = 7.3 Hz, 2H), 7.39 (td, *J* = 7.3, 0.7 Hz, 2H), 7.56 (d, *J* = 7.3 Hz, 2H); ^{13}C NMR (150 MHz, $CDCl_3$) δ 20.3, 21.3, 114.7, 117.8, 120.0, 123.9, 128.4, 128.8, 128.9, 133.4, 134.3, 134.4, 135.0, 136.0, 138.4, 141.0, 143.6, 146.4, 146.5, 157.4, 193.3; HRMS (APCI) *m/z* calcd for $C_{48}H_{34}O_2$ $[M]^-$: 642.2553, found: 642.2559.

Synthesis of 1a

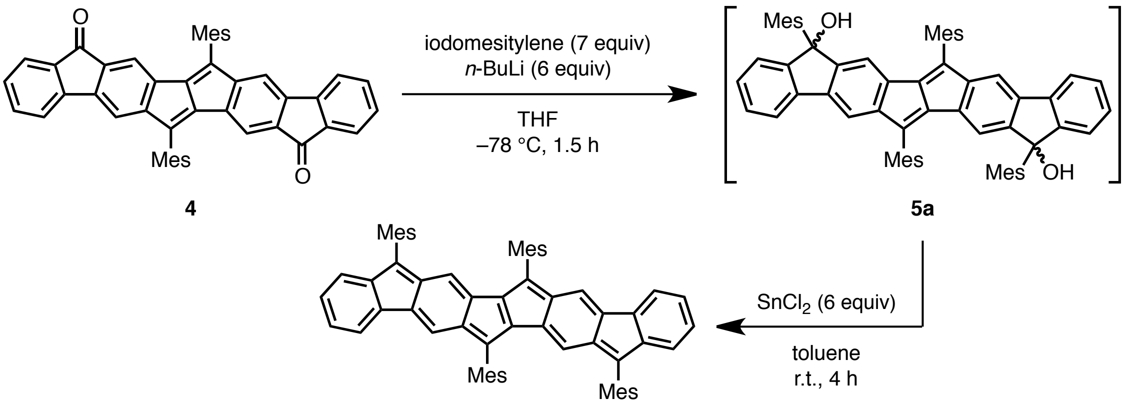

To a Schlenk tube containing a magnetic stirring bar were added iodomesitylene (322 mg, 1.20 mmol) and dry THF (7 mL). A solution of *n*-butyllithium in hexane (625 μL, 1.6 M, 1.0 mmol) was added at –78 °C. After stirring the mixture at –78 °C for 30 min, a suspension of **4** (109 mg, 170 μmol) in THF (17 mL) was added, and the resultant mixture was stirred at –78 °C for 30 min, then further stirred at room temperature for 1 hour, the mixture was quenched with saturated NH_4Cl aqueous solution, extracted with EtOAc, dried over with Na_2SO_4, and concentrated under reduced pressure to afford crude mixture containing **5a**.

To a 50 mL round bottom flask were added a magnetic stirring bar, the crude mixture and $SnCl_2$ (193 mg, 1.00 mmol). Contents were evacuated, then back-filled with nitrogen gas three times, then dry toluene (10 mL) was added. The resulting mixture was stirred at room temperature for 4 hours then passed the short silica-gel plug, eluted with CH_2Cl_2. The solvents were evaporated under reduced pressure to afford the crude reaction mixture. The crude reaction mixture was purified by silica gel column chromatography (hexane/CH_2Cl_2 = 1:1) to afford product **1a** as a deep green solid (23.0 mg, 16%). For X-ray crystallography, the product was recrystallized in CS_2/Et_2O.

^{1}H NMR (600 MHz, CS_2/CD_2Cl_2, room temperature) δ 2.27 (br), 2.38 (br), 6.84 (br): HRMS (APCI) *m*/*z* calcd for $C_{66}H_{56}$ [M]$^-$: 848.4377, found: 848.4385.

Synthesis of 1b

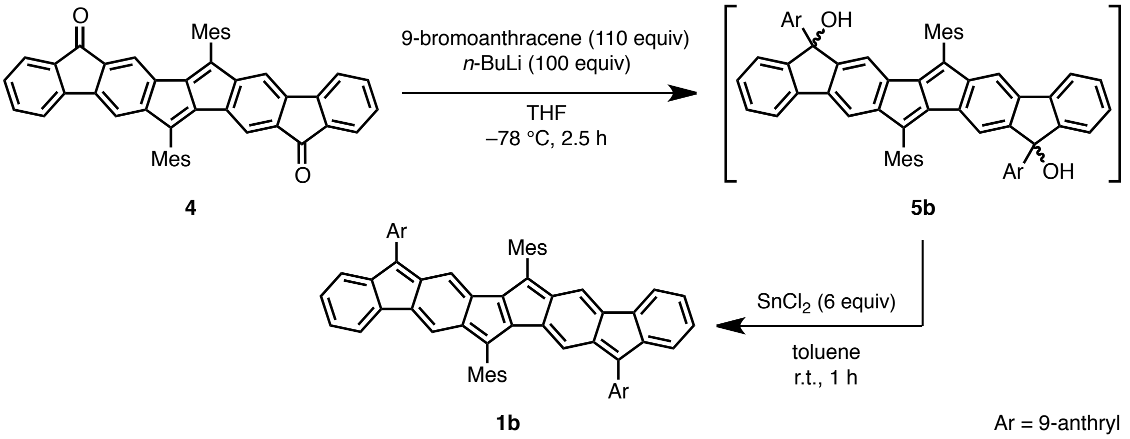

To a Schlenk tube containing a magnetic stirring bar were added 9-bromoanthracene (833 mg, 3.20 mmol) and dry THF (10 mL). A solution of *n*-butyllithium in hexane (1.8 mL, 1.6 M, 2.9 mmol) was added at –78 °C. After stirring the mixture at –78 °C for 30 min, a suspension of **4** (19.0 mg, 29.0 μmol) in THF (5 mL) was added, and the resultant mixture was stirred at –78 °C for 30 min, then further stirred at room temperature for 2 hours, the mixture was quenched with saturated NH_4Cl aqueous solution, extracted with Et_2O, dried over with Na_2SO_4, and concentrated under reduced pressure to afford crude mixture containing **5b**. The unreacted starting materials were removed from crude mixture by silica-gel column chromatography (hexane/CH_2Cl_2 = 2:1).

To a Schlenk tube were added a magnetic stirring bar, the crude mixture, and $SnCl_2$ (33.0 mg, 174 μmol). Contents were evacuated, then back-filled with nitrogen gas three times, then dry toluene (10 mL) was added. The resulting mixture was stirred at room temperature for 1 hour then passed the short silica-gel plug, eluted with CH_2Cl_2. The solvents were evaporated under reduced pressure to afford the crude reaction mixture. The crude reaction mixture was purified by silica-gel column chromatography (hexane/CH_2Cl_2 = 1:1) to afford product **1b** as deep green solid (8.4 mg, 30%). For X-ray crystallography, the product was recrystallized in CS_2/Et_2O.

^{1}H NMR (600 MHz, $CDCl_3$) δ 2.00 (br), 2.27 (br), 6.39 (br): HRMS (APCI) *m/z* calcd for $C_{76}H_{52}$ $[M]^-$: 964.4061, found: 964.4064.

2. X-ray crystallography

Details of the crystal data and a summary of the intensity data collection parameters for obtained products are listed in below table. In each case, a suitable crystal was mounted with mineral oil on a glass fiber and transferred to the goniometer of a Rigaku PILATUS or Saturn CCD diffractometer. Graphite-monochromated Mo Kα radiation (λ = 0.71075 Å) was used. The structures were solved by direct methods with (SIR-97)[S2] and refined by full-matrix least-squares techniques against F^2 (SHELXL-97).[S3] The intensities were corrected for Lorentz and polarization effects. The non-hydrogen atoms were refined anisotropically. Hydrogen atoms were placed using AFIX instructions (Table S1).

Table S1. Crystallographic data and structure refinement details for products.

	**1a**	**1b**·Et_2O
formula	C66H56	C84H72O2
fw	849.11	1113.42
T (K)	103(2)	103(2)
λ 2)(	0.71075	0.71075
cryst syst	Trigonal	Monoclicnic
space group	R-3	$P2_1/n$
a (Å)	37.115(8)	14.623(6)
b (Å)	37.115(8)	14.269(5)
c (Å)	8.9290(19)	15.120(6)
α (deg)	90	90
β (deg)	90	109.197(7)
γ (deg)	120	90
V (Å^3)	10652(4)	2979(2)
Z	9	2
D_{calc}, (g / cm^3)	1.191	1.241
μ (mm^{-1})	0.067	0.072
F(000)	4068	1184
cryst size (mm)	0.10 × 0.05 × 0.03	0.10 × 0.07 × 0.03
2θ range, (deg)	3.23–25.00	3.10–25.00
reflns collected	24070	21501
indep reflns/R_{int}	4163/0.0741	5230/0.1878
params	304	393
GOF on F^2	1.059	0.957
R_1, wR_2 [I>2σ(I)]	0.0560, 0.1134	0.0960, 0.2429
R_1, wR_2 (all data)	0.0951, 0.1371	0.1713, 0.2959

S2) A. Altomare, M. C. Burla, M. Camalli, G. L. Cascarano, C. Giacovazzo, A. Guagliardi, A. G. G. Moliterni, G. Polidori and R. Spagna, *J. Appl. Crystallogr.*, 1999, **32,** 115–119.
S3) G. M. Sheldrick, University of Göttingen: Göttingen, Germany, 1997.

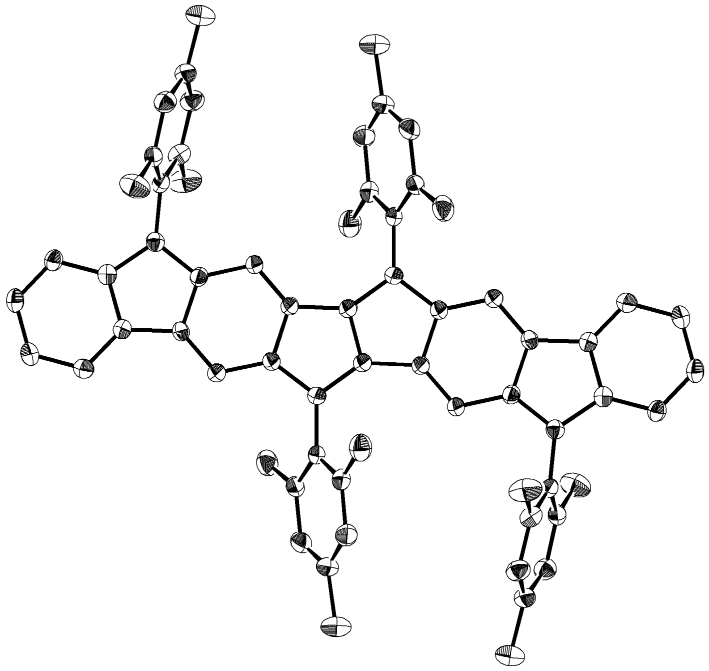

Figure S1. ORTEP drawing of **1a** with 50% thermal probability. All hydrogen atoms are omitted for clarity. Half of the entire structure constitutes an asymmetric unit.

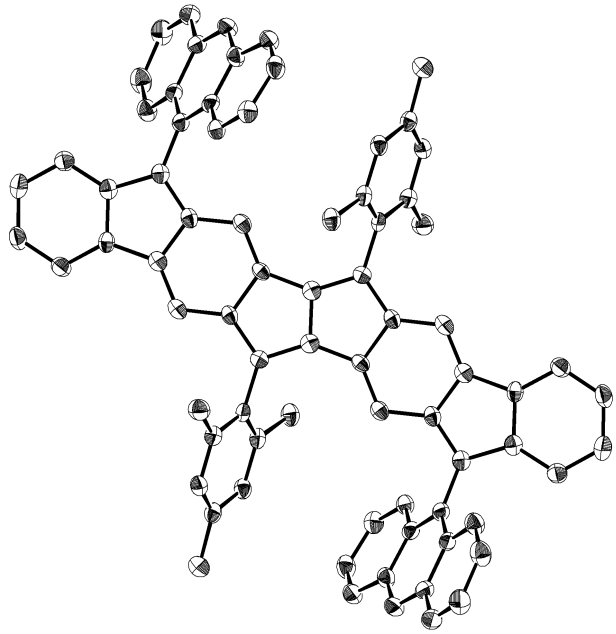

Figure S2. ORTEP drawing of **1b** with 50% thermal probability. Solvent molecule and all hydrogen atoms are omitted for clarity. A half of the entire structure constitutes an asymmetric unit.

Table S2. Selected bond lengths (Å) of **1a,b** and optimized PDFs.

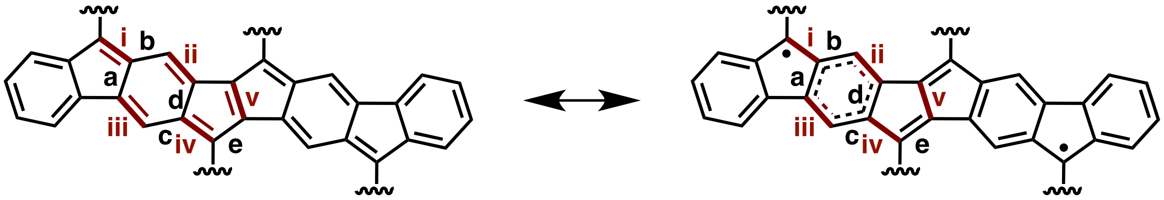

	X-ray		(U)B3LYP/6-311+G(d,p)		
	**1a**	**1b**	**c-PDF**	**s-PDF**	**t-PDF**
**i**	1.401(3)	1.416(6)	1.388	1.410	1.410
**ii**	1.363(3)	1.364(6)	1.370	1.378	1.381
**iii**	1.372(3)	1.374(7)	1.374	1.388	1.390
**iv**	1.424(3)	1.420(6)	1.407	1.445	1.457
**v**	1.407(5)	1.415(9)	1.417	1.453	1.476
**a**	1.443(3)	1.432(6)	1.457	1.444	1.446
**b**	1.417(3)	1.424(6)	1.430	1.419	1.417
**c**	1.404(3)	1.418(7)	1.420	1.403	1.400
**d**	1.459(3)	1.452(6)	1.468	1.450	1.450
**e**	1.413(3)	1.420(7)	1.413	1.379	1.366

3. VT NMR measurement of 1a

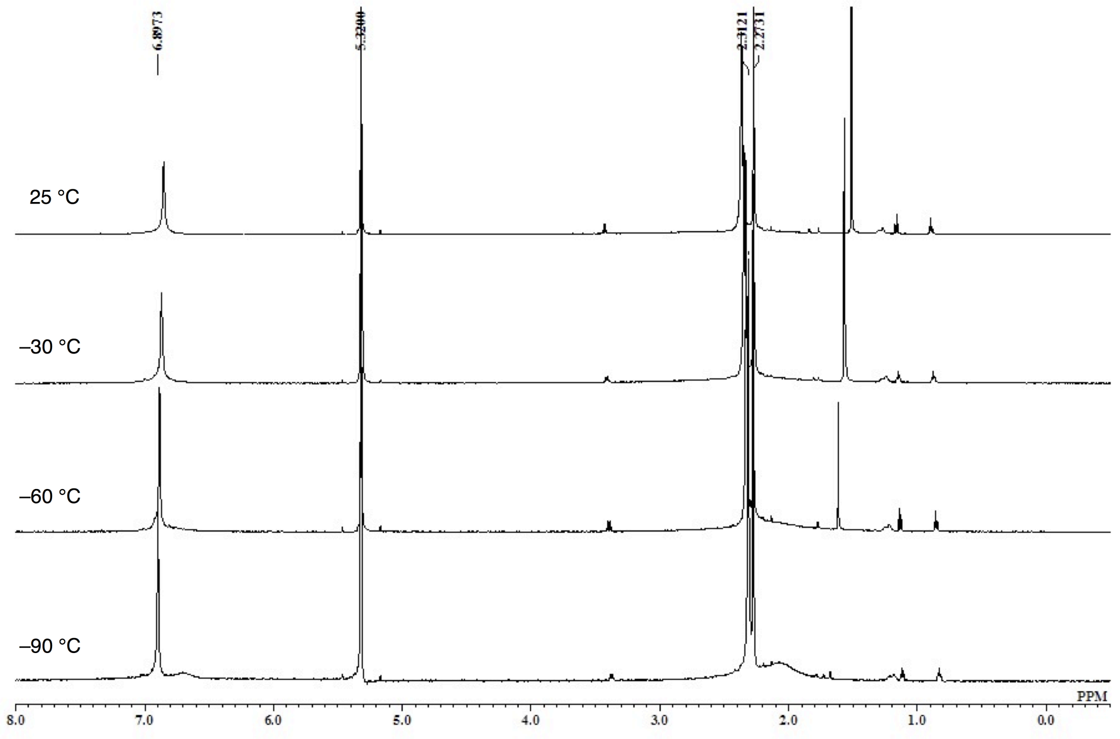

Figure S3. Variable Temperature ^{1}H NMR spectra of **1a** (CD_2Cl_2/CS_2)

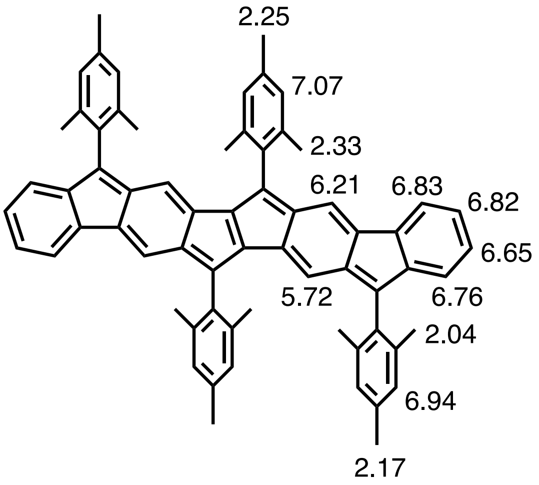

Figure S4. ^{1}H NMR chemical shift (ppm) of **1a** calculated by GIAO UB3LYP/6-311+G(2d,p)// UB3LYP/6-31G(d).

4. UV–vis–NIR absorption spectroscopy

UV–vis–NIR absorption spectra were recorded on a SHIMADZU UV-3600 spectrometer with a resolution of 2.0 nm and 1 cm quartz cell.

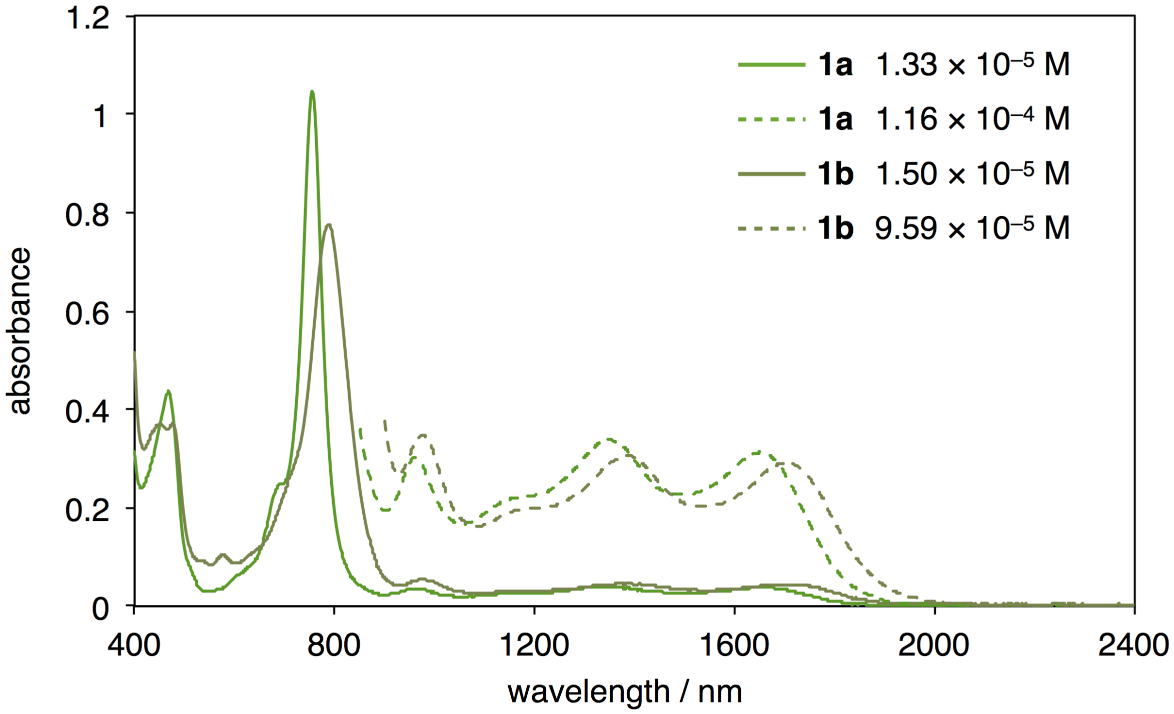

Figure S5. UV-vis-NIR spectra of CS_2 solution of **1a** and **1b** in each two concentrations.

Table S3. Absorption maxima (nm).

**1a**	**1b**
756	786
962	976
1348	1380
1652	1702

5. Cyclic voltammetry

Cyclic voltammetry was performed by BAS ALS-600D Electrochemical Analyzer.

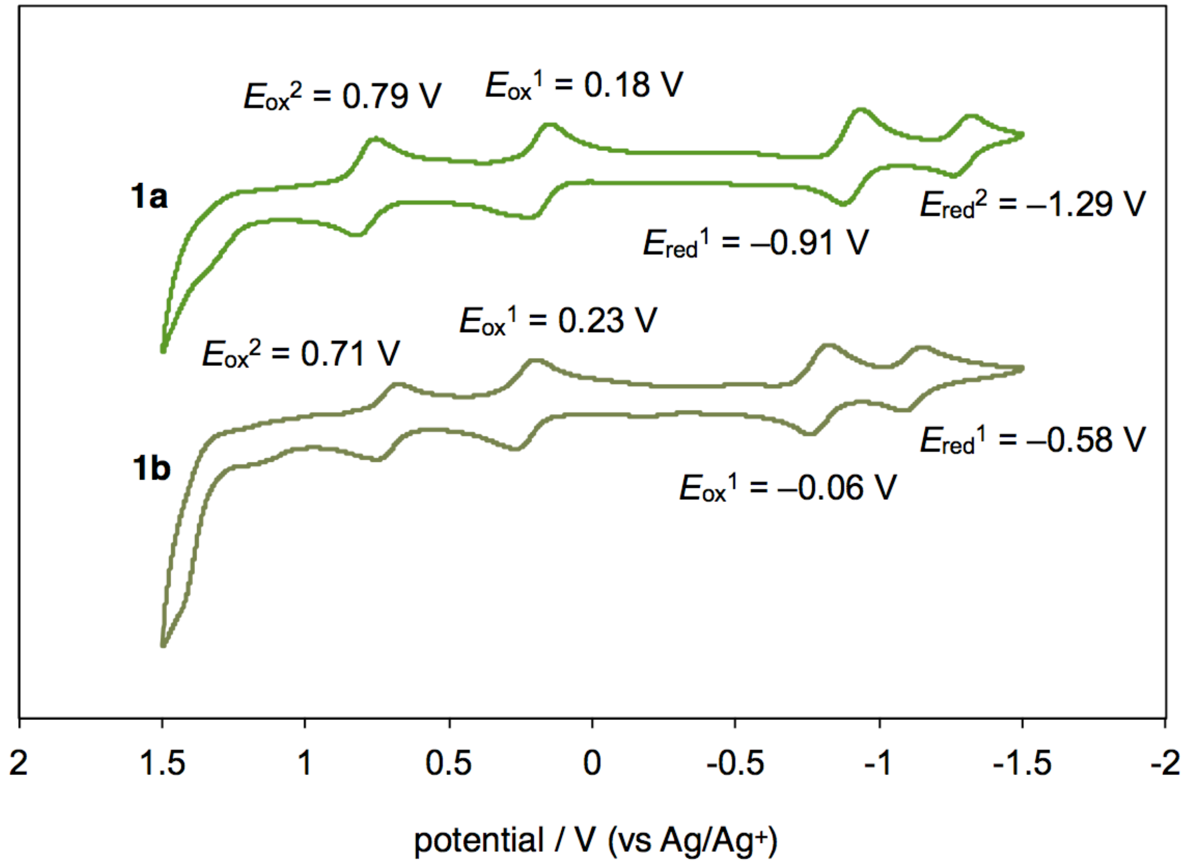

Figure S6. Cyclic voltammograms of **1a** and **1b** with redox potentials.

6. SQUID measurement

Temperature dependence of magnetization was measured under 10000 Oe using SQUID (Quantum design, MPMS). Powder of **1a** (11.1 mg) was used. Fitting of the data was done by using Bleaney–Bowers equation.[S4] Rise of magnetization below 200 K may be due to small amount of doublet impurity.

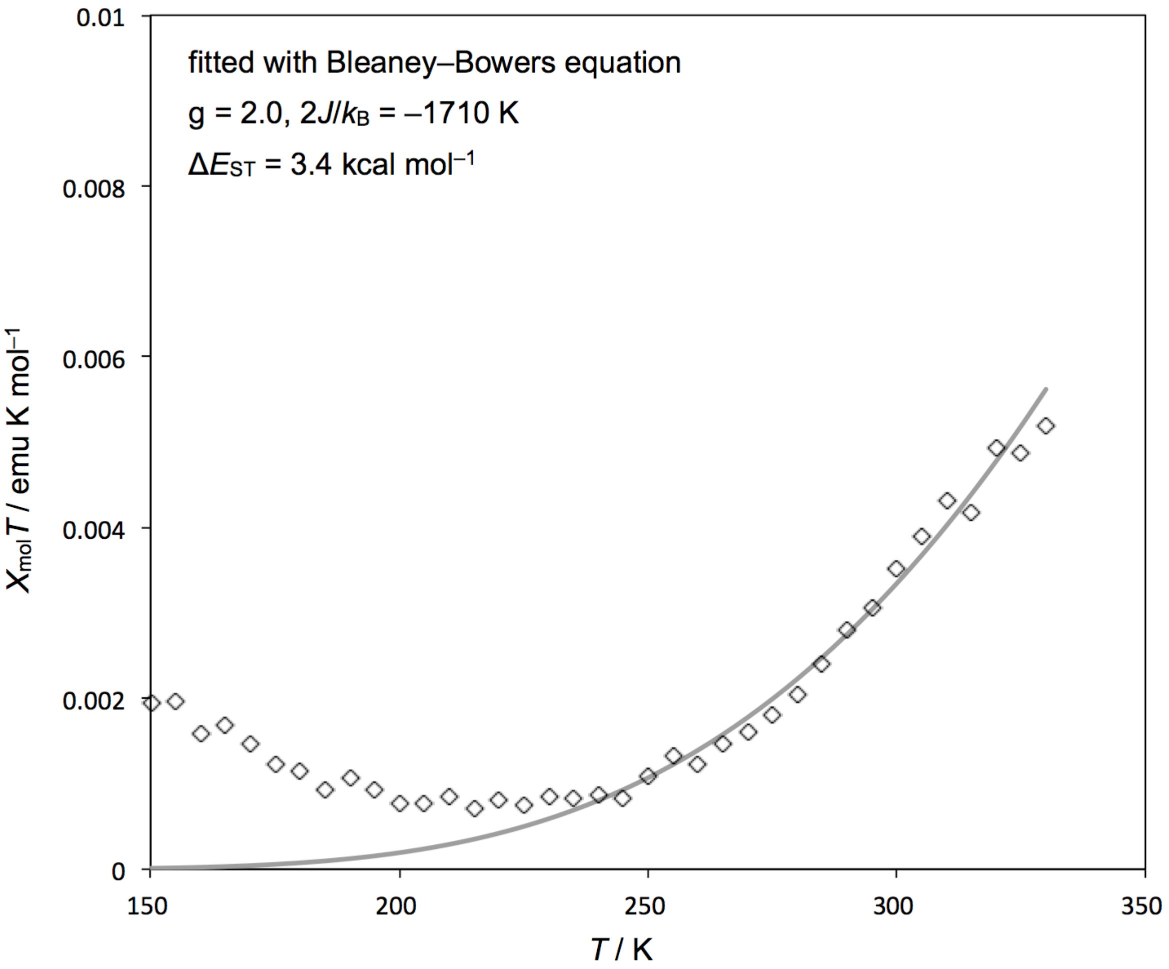

Figure S7. Temperature dependence of magnetization of **1a** in solid state.

S4) Bleaney, B.; Bowers, K. D. *Proc. R. Soc. London Ser. A* **1952**, *214*, 451.

7. Computational Study

The Gaussian 09 program[S5] running on a SGI Altix4700 system was used for optimization. Structures were optimized without any symmetry assumptions. Calculation of singlet open-shell was performed followed by previously reported method.[S6] The initial geometry optimization of pentaleno[1,2-*b*:4,5-*b*']difluorene (PDF) was performed with the restricted (e.g. B3LYP) level of theory.[S7] The resulting singlet closed-shell structure of PDF (**c-PDF**) was further tested for its stability with the stable=opt keyword.[S8] Then the Guess=Read keyword was used to perform the optimization at the unrestricted (e.g. UB3LYP) level (singlet open-shell, **s-PDF**). Visualization of the results was performed by use of GaussView 5.0.9 software.

Table S4. Relative energy values of the three possible electronic states of PDFs (kcal mol^{-1}).

	(U)B3LYP/6-311+G(d,p)	(U)CAM-B3LYP/6-311+G(d,p)	(U)M06-2X/6-311+G(d,p)
**c-PDF**	2.0	8.0	3.2
**sb-PDF**	0.0	0.0	0.0
**t-PDF**	1.4	–0.1	0.6

S5) M. J. Frisch, G. W. Trucks, H. B. Schlegel, G. E. Scuseria, M. A. Robb, J. R. Cheeseman, G. Scalmani, V. Barone, B. Mennucci, G. A. Petersson, H. Nakatsuji, M. Caricato, X. Li, H. P. Hratchian, A. F. Izmaylov, J. Bloino, G. Zheng, J. L. Sonnenberg, M. Hada, M. Ehara, K. Toyota, R. Fukuda, J. Hasegawa, M. Ishida, T. Nakajima, Y. Honda, O. Kitao, H. Nakai, T. Vreven, J. A. Montgomery, Jr., J. E. Peralta, F. Ogliaro, M. Bearpark, J. J. Heyd, E. Brothers, K. N. Kudin, V. N. Staroverov, T. Keith, R. Kobayashi, J. Normand, K. Raghavachari, A. Rendell, J. C. Burant, S. S. Iyengar, J. Tomasi, M. Cossi, N. Rega, J. M. Millam, M. Klene, J. E. Knox, J. B. Cross, V. Bakken, C. Adamo, J. Jaramillo, R. Gomperts, R. E. Stratmann, O. Yazyev, A. J. Austin, R. Cammi, C. Pomelli, J. W. Ochterski, R. L. Martin, K. Morokuma, V. G. Zakrzewski, G. A. Voth, P. Salvador, J. J. Dannenberg, S. Dapprich, A. D. Daniels, O. Farkas, J. B. Foresman, J. V. Ortiz, J. Cioslowski and D. J. Fox, *Gaussian 09, Revision C.01,* Gaussian, Inc., Wallingford CT, 2010.

S6) Z. Sun, K.-W. Huang and J. Wu, *J. Am. Chem. Soc.* 2011, **133**, 11896.

S7) (a) A. D. Becke, *J. Chem. Phys.* 1993, **98**, 5648–5652; (b) C. Lee, W. Yang and R. G. Parr, *Phys. Rev. B* 1988, **37**, 785–789; (c) T. Yanai, D. Tew and N. Handy, *Chem. Phys. Lett.* 2004, **393**, 51.

S8) R. Seeger and J. Pople, A. *J. Chem. Phys.* 1977, **66**, 3045.

Table S4. Cartesian coordinates for the conformations by using (U)B3LYP/6-311+G(d,p) level.

c-PDF E = -1152.36685574 Hartree

C	5.601643	-1.653091	0.000000	C	-1.378774	1.143601	0.000000	H	6.799566	-4.857988	0.000000
C	4.200942	-1.219854	0.000000	C	-2.193973	-0.084037	0.000000	H	8.934602	-3.599117	0.000000
C	3.393477	-2.438208	0.000000	C	-1.353906	-1.172768	0.000000	H	8.933877	-1.138323	0.000000
C	4.219637	-3.521950	0.000000	C	-1.956704	2.364142	0.000000	H	6.811401	0.127574	0.000000
C	5.602254	-3.067411	0.000000	C	-3.393477	2.438208	0.000000	H	4.213779	0.912528	0.000000
C	6.795632	-3.773975	0.000000	C	-4.200942	1.219854	0.000000	H	1.369837	-3.276544	0.000000
C	7.992576	-3.064260	0.000000	C	-3.623181	-0.002189	0.000000	H	1.668193	2.207852	0.000000
C	7.991456	-1.672938	0.000000	C	-4.219637	3.521950	0.000000	H	-1.668193	-2.207852	0.000000
C	6.795632	-0.956294	0.000000	C	-5.602254	3.067411	0.000000	H	-1.369837	3.276544	0.000000
C	3.623181	0.002189	0.000000	C	-5.601643	1.653091	0.000000	H	-4.213779	-0.912528	0.000000
C	2.193973	0.084037	0.000000	C	-6.795632	3.773975	0.000000	H	-3.909476	4.558888	0.000000
C	1.378774	-1.143601	0.000000	C	-7.992576	3.064260	0.000000	H	-6.799566	4.857988	0.000000
C	1.956704	-2.364142	0.000000	C	-7.991456	1.672938	0.000000	H	-8.934602	3.599117	0.000000
C	1.353906	1.172768	0.000000	C	-6.795632	0.956294	0.000000	H	-8.933877	1.138323	0.000000
C	0.002920	0.691248	0.000000	H	3.909476	-4.558888	0.000000	H	-6.811401	-0.127574	0.000000
C	-0.002920	-0.691248	0.000000								

sb-PDF E = -1152.37955342 Hartree, S^2 = 1.1224, NOON of LUNO = 0.54869

C	5.535733	-1.810769	0.000000	C	-1.361612	1.202798	0.000000	H	6.746492	-5.016844	0.000000
C	4.137258	-1.373753	0.000000	C	-2.178507	0.025222	0.000000	H	8.875224	-3.750741	0.000000
C	3.327783	-2.549790	0.000000	C	-1.312467	-1.147815	0.000000	H	8.867450	-1.289778	0.000000
C	4.173077	-3.681171	0.000000	C	-1.919723	2.454370	0.000000	H	6.740951	-0.028791	0.000000
C	5.535733	-3.231017	0.000000	C	-3.327783	2.549790	0.000000	H	4.178828	0.782637	0.000000
C	6.738744	-3.932864	0.000000	C	-4.137258	1.373753	0.000000	H	1.306122	-3.348329	0.000000
C	7.931021	-3.219750	0.000000	C	-3.567133	0.112933	0.000000	H	1.672817	2.167993	0.000000
C	7.926362	-1.826536	0.000000	C	-4.173077	3.681171	0.000000	H	-1.672817	-2.167993	0.000000
C	6.727309	-1.112770	0.000000	C	-5.535733	3.231017	0.000000	H	-1.306122	3.348329	0.000000
C	3.567133	-0.112933	0.000000	C	-5.535733	1.810769	0.000000	H	-4.178828	-0.782637	0.000000
C	2.178507	-0.025222	0.000000	C	-6.738744	3.932864	0.000000	H	-3.850157	4.713415	0.000000
C	1.361612	-1.202798	0.000000	C	-7.931021	3.219750	0.000000	H	-6.746492	5.016844	0.000000
C	1.919723	-2.454370	0.000000	C	-7.926362	1.826536	0.000000	H	-8.875224	3.750741	0.000000
C	1.312467	1.147815	0.000000	C	-6.727309	1.112770	0.000000	H	-8.867450	1.289778	0.000000
C	0.021947	0.727463	0.000000	H	3.850157	-4.713415	0.000000	H	-6.740951	0.028791	0.000000
C	-0.021947	-0.727463	0.000000								

t-PDF E = -1152.37974655 Hartree, S^2 = 2.2039

C	5.526467	-1.825022	0.000000	C	-1.350731	1.214449	0.000000	H	6.738803	-5.026162	0.000000
C	4.125804	-1.389070	0.000000	C	-2.171584	0.036369	0.000000	H	8.865600	-3.756810	0.000000
C	3.314135	-2.569763	0.000000	C	-1.312045	-1.146713	0.000000	H	8.857047	-1.296593	0.000000
C	4.159100	-3.696047	0.000000	C	-1.906766	2.469433	0.000000	H	6.725558	-0.039376	0.000000
C	5.526467	-3.243291	0.000000	C	-3.314135	2.569763	0.000000	H	4.173720	0.766858	0.000000
C	6.728231	-3.942245	0.000000	C	-4.125804	1.389070	0.000000	H	1.290175	-3.361177	0.000000
C	7.921230	-3.225889	0.000000	C	-3.559767	0.127034	0.000000	H	1.681586	2.163403	0.000000
C	7.916843	-1.834705	0.000000	C	-4.159100	3.696047	0.000000	H	-1.681586	-2.163403	0.000000
C	6.714814	-1.123459	0.000000	C	-5.526467	3.243291	0.000000	H	-1.290175	3.361177	0.000000
C	3.559767	-0.127034	0.000000	C	-5.526467	1.825022	0.000000	H	-4.173720	-0.766858	0.000000
C	2.171584	-0.036369	0.000000	C	-6.728231	3.942245	0.000000	H	-3.839116	4.729045	0.000000
C	1.350731	-1.214449	0.000000	C	-7.921230	3.225889	0.000000	H	-6.738803	5.026162	0.000000
C	1.906766	-2.469433	0.000000	C	-7.916843	1.834705	0.000000	H	-8.865600	3.756810	0.000000
C	1.312045	1.146713	0.000000	C	-6.714814	1.123459	0.000000	H	-8.857047	1.296593	0.000000
C	0.024733	0.736274	0.000000	H	3.839116	-4.729045	0.000000	H	-6.725558	0.039376	0.000000
C	-0.024733	-0.736274	0.000000								

8. ^{1}H NMR and ^{13}C NMR spectra of new compounds

^{1}H NMR spectrum of **9** (600 MHz, $CDCl_3$)

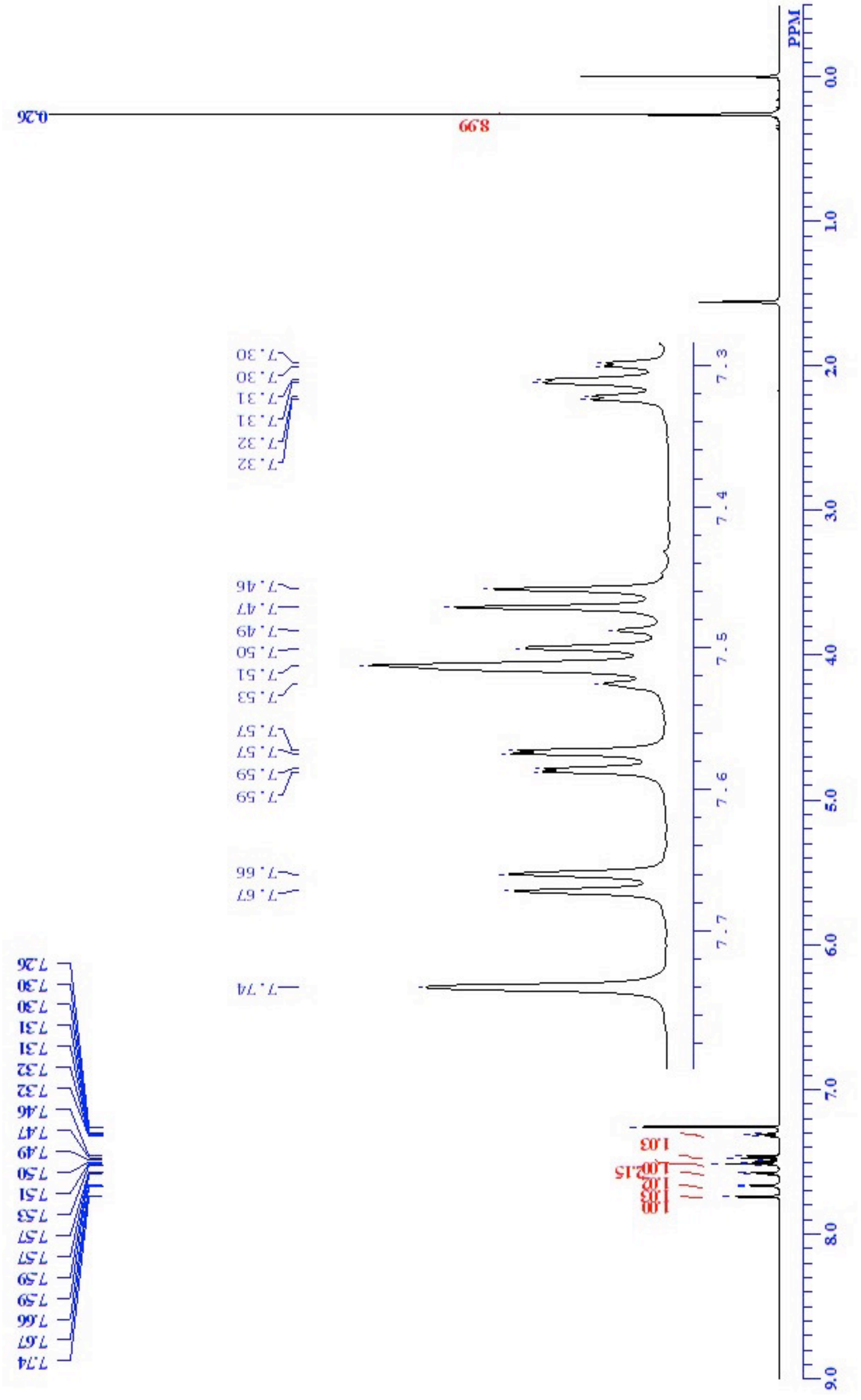

^{13}C NMR spectrum of **9** (600 MHz, $CDCl_3$)

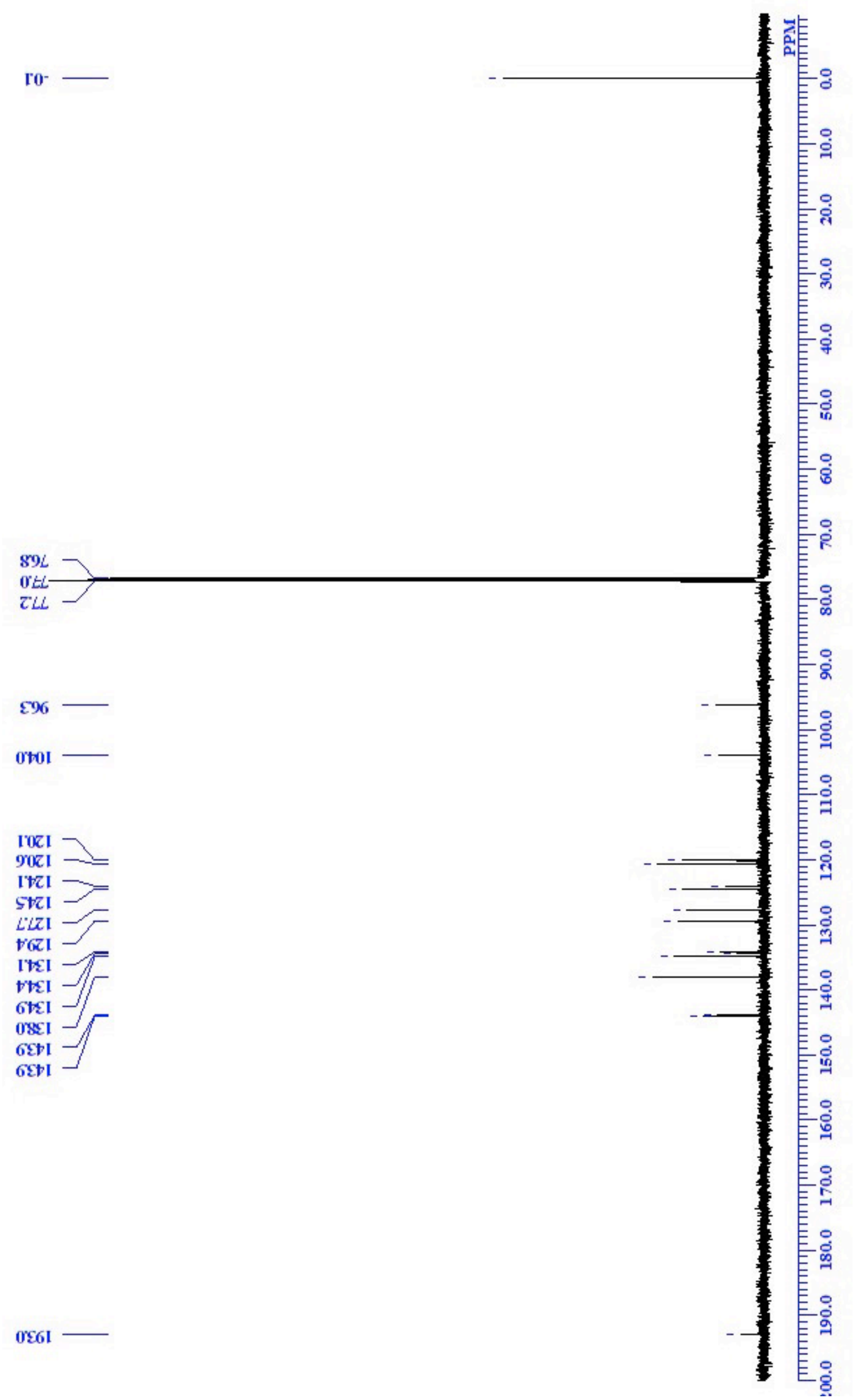

^{1}H NMR spectrum of **3** (600 MHz, $CDCl_3$)

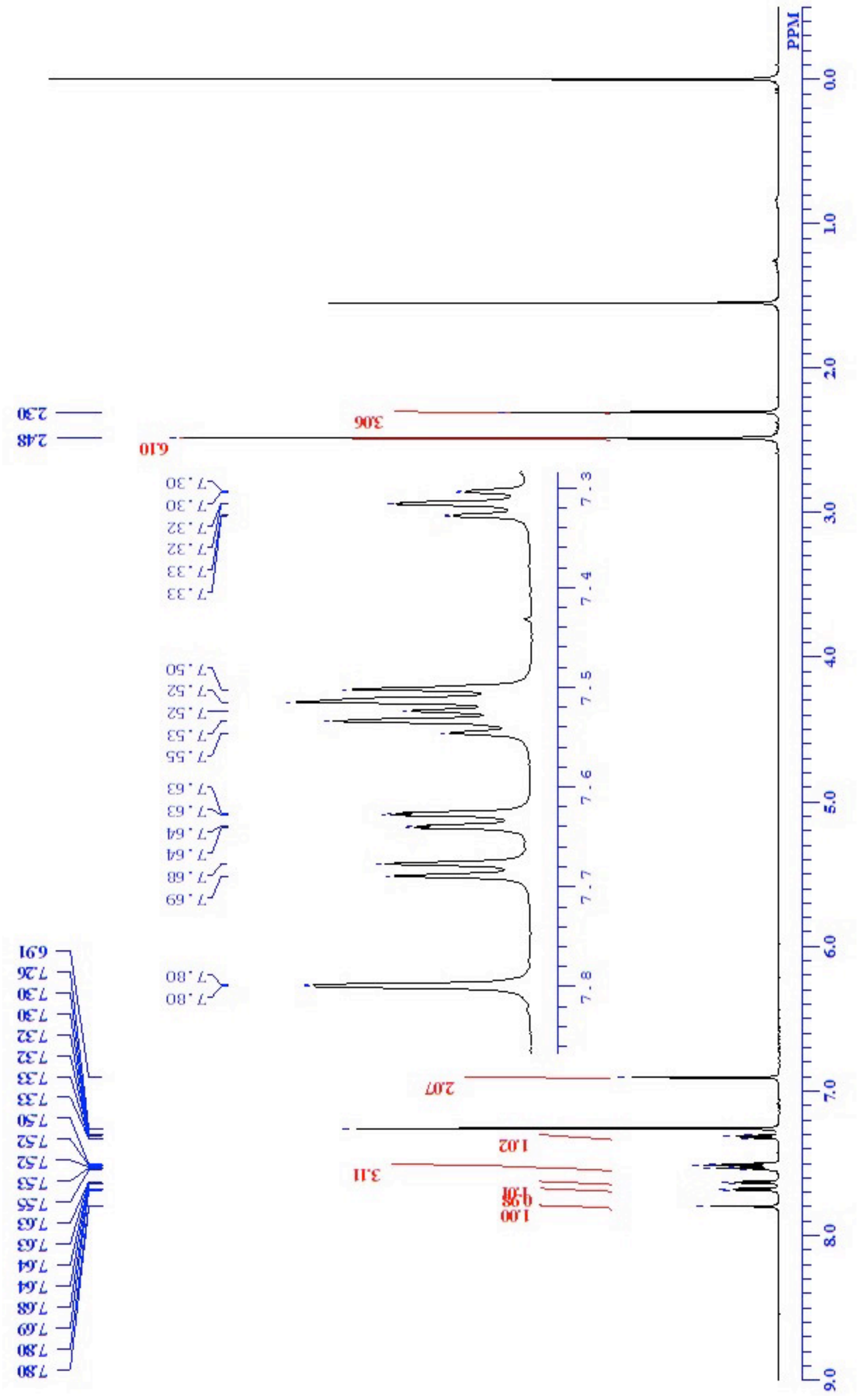

^{13}C NMR spectrum of **3** (600 MHz, $CDCl_3$)

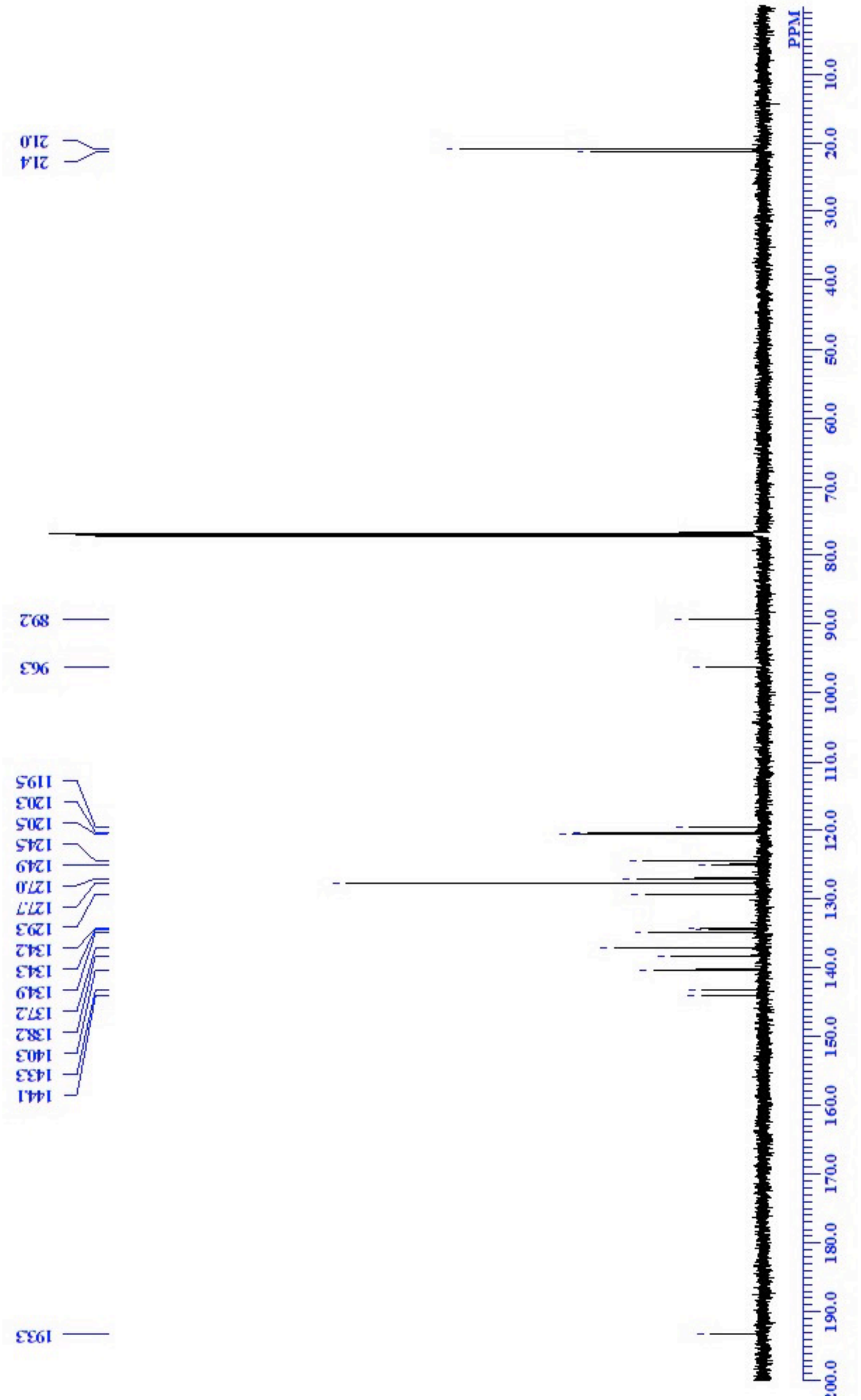

^{1}H NMR spectrum of **4** (600 MHz, $CDCl_3$)

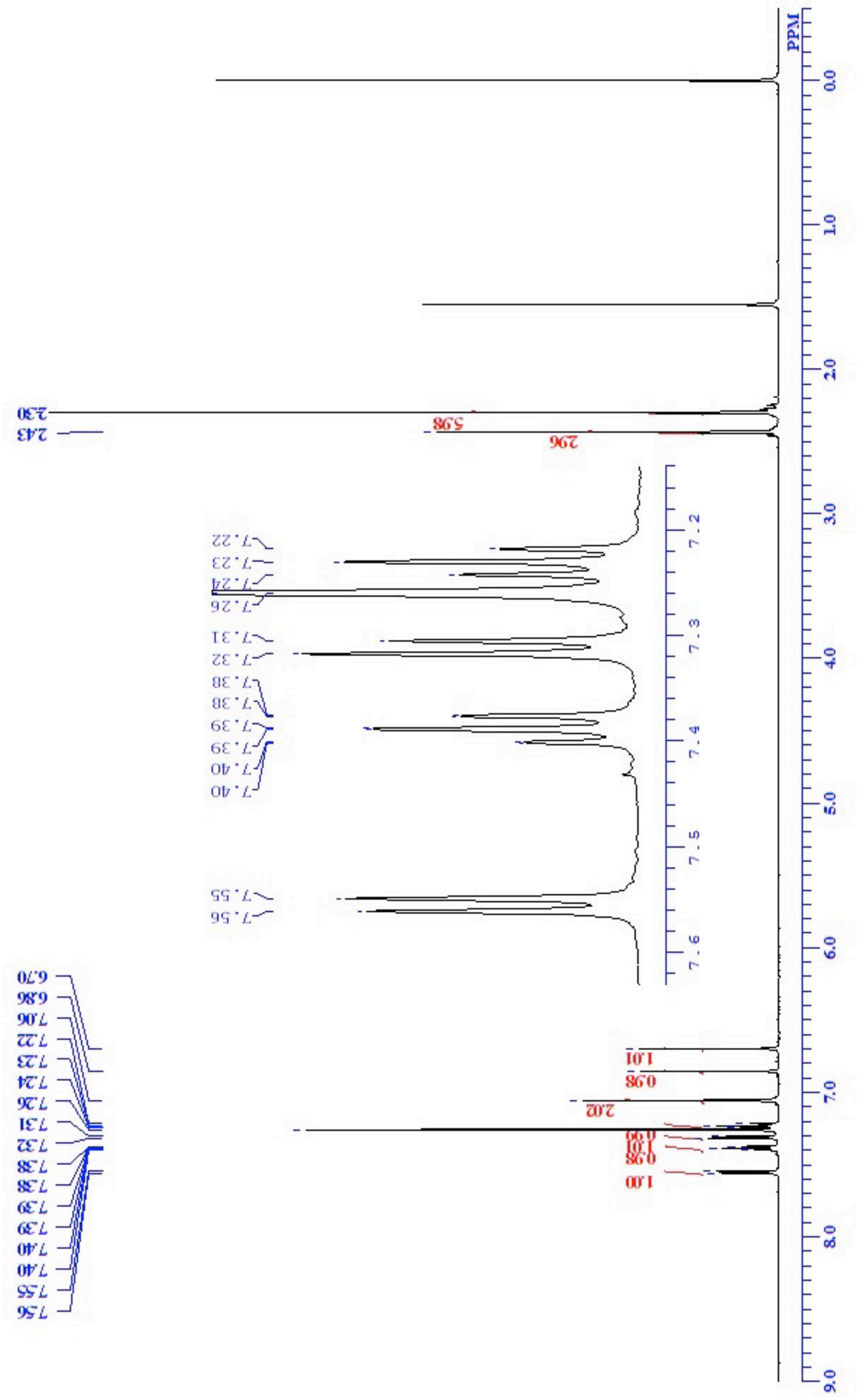

^{13}C NMR spectrum of **4** (600 MHz, $CDCl_3$)

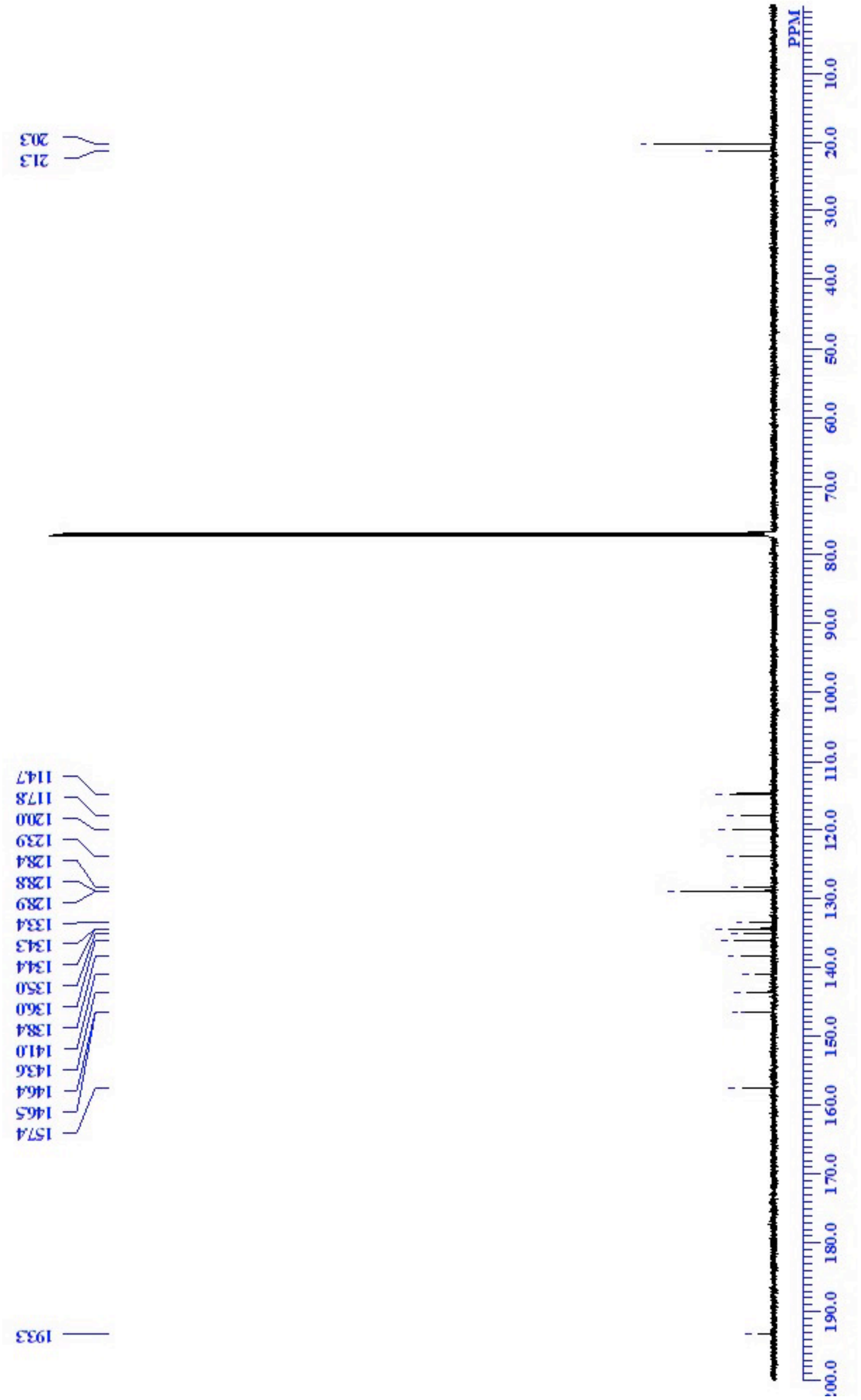

^{1}H NMR spectrum of **1a** (600 MHz, CD_2Cl_2/CS_2)

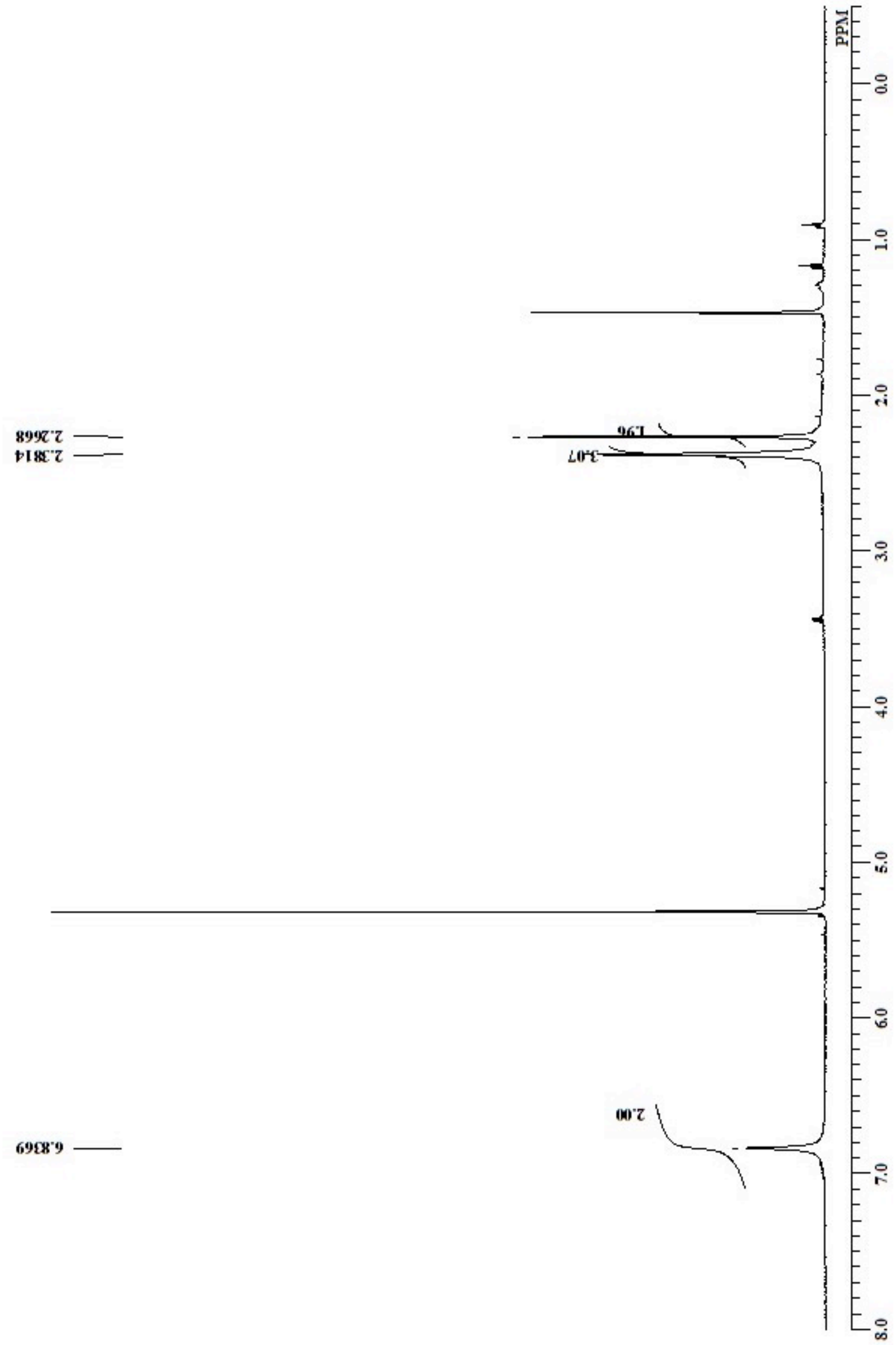

^{1}H NMR spectrum of **1b** (600 MHz, $CDCl_3$)

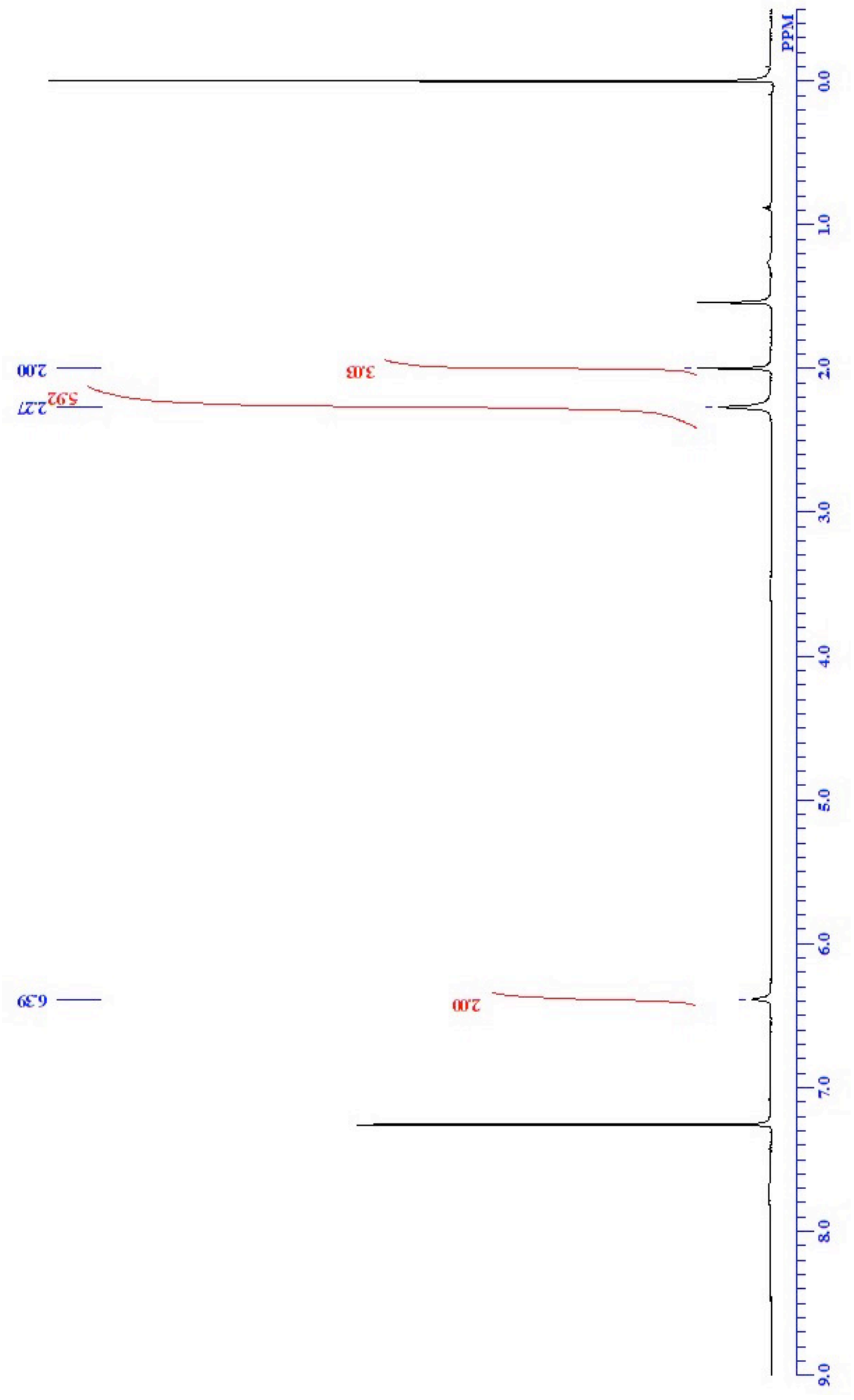

Supplement: Supplementary file 1 [file SC-007-C5SC03391H-s001.pdf]
